# Reduced Representation and Whole‐Genome Sequencing Approaches Highlight Beluga Whale Populations Associated to Eastern Canada Summer Aggregations

**DOI:** 10.1111/eva.70058

**Published:** 2024-12-18

**Authors:** Luca Montana, Trevor T. Bringloe, Audrey Bourret, Caroline Sauvé, Arnaud Mosnier, Steven H. Ferguson, Lianne Postma, Véronique Lesage, Cortney A. Watt, Mike O. Hammill, Geneviève J. Parent

**Affiliations:** ^1^ Maurice Lamontagne Institute Fisheries and Oceans Canada Mont‐Joli Quebec Canada; ^2^ Freshwater Institute Fisheries and Oceans Canada Winnipeg Manitoba Canada; ^3^ Department of Biological Sciences University of Manitoba Winnipeg Manitoba Canada

**Keywords:** cetacean, conservation, ddRADseq, dispersal, management, migration, nuclear DNA, whole genome sequencing

## Abstract

Effective conservation strategies inherently depend on preserving populations, which in turn requires accurate tools for their detection. Beluga whales ( *Delphinapterus leucas* ) inhabit the circumpolar Arctic and form discrete summer aggregations. Previous genetic studies using mitochondrial and microsatellite loci have delineated distinct populations associated to summer aggregations but the extent of dispersal and interbreeding among these populations remains largely unknown. Such information is essential for the conservation of populations in Canada as some are endangered and harvested for subsistence by Inuit communities. Here, we used reduced representation and whole‐genome sequencing approaches to characterize population structure of beluga whales in eastern Canada and examine admixture between populations. A total of 905 beluga whales sampled between 1989 and 2021 were genotyped. Six main genomic clusters, with potential subclusters, were identified using multiple proxies for population structure. Most of the six main genomic clusters were consistent with previously identified populations, except in southeast Hudson Bay where two clusters were identified. Beluga summer aggregations may consequently be comprised of more than one distinct population. A low number of dispersers were identified between summer aggregations and limited interbreeding was detected between the six genomic clusters. Our work highlights the value of genomic approaches to improve our understanding of population structure and reproductive behavior in beluga whales, offering insights applicable to other cetacean species of conservation concern. An expansion of the geographical scope and increase in number of genotyped individuals will, however, be needed to improve the characterization of the finer scale structure and of the extent of admixture between populations.

## 1 Introduction

Many cetaceans have experienced severe declines in abundance over the last two centuries due to anthropogenic disturbances such as harvest, fisheries bycatch, pollution, and habitat degradation. For cetaceans with wide distributions, the impact of these stressors varies spatially, resulting in differential consequences among aggregations. Some species have rebounded from low numbers while others have shown little sign of recovery since conservation strategies were implemented (Albouy et al. 2020; Davidson et al. 2012; Nelms et al. 2021; Schipper et al. 2008). The extent of species distribution and spatial heterogeneity may partially explain the relative success of conservation strategies. In particular, characterizing spatial distribution of aggregations and the demographic links, that is, dispersal and interbreeding, between aggregations can inform, guide, and optimize conservation strategies.

Beluga whales ( *Delphinapterus leucas* ) are considered of Least Concern globally under the International Union for Conservation of Nature (IUCN) Red List of Threatened Species given their total abundance of approximately 200,000 animals (Lowry, Reeves, and Laidre 2017). However, this species is of particular interest for conservation efforts with a discontinuous circumpolar distribution and multiple populations considered at risk of extinction (Hobbs et al. 2019; Lowry, Reeves, and Laidre 2017; Richard 2014). A recent review identified a total of 21 management units based on non‐overlapping summer aggregations and genetic analyses (Hobbs et al. 2019). Only seven have known abundance trends, with half of these declining (Hammill et al. 2023; Hobbs et al. 2019).

The Committee on the Status of Endangered Wildlife in Canada (COSEWIC) recognizes seven beluga units in eastern Canada: (1) Eastern High Arctic‐Baffin Bay, (2) Cumberland Sound, (3) Ungava Bay, (4) Western Hudson Bay, (5) Eastern Hudson Bay, (6) James Bay, and (7) St. Lawrence Estuary (COSEWIC 2014, 2016, 2020). COSEWIC refers to these as designatable units (DUs) based on cultural or genetic distinctiveness, which each are units of biodiversity that would be irreplaceable in case of extinction or if extirpated from a jurisdiction (Green 2005). All seven eastern Canada DUs were depleted due to commercial harvesting that lasted until the mid‐20th century (Heide‐Jørgensen and Reeves 1996; Reeves and Mitchell 1987). Four beluga DUs continue to have low abundance and have not shown signs of recovery, in spite of the implementation of management plans. They include Cumberland Sound (*N* = 1200; Watt et al. 2021), Ungava Bay (*N* = 70; Sauvé et al. 2023), Eastern Hudson Bay (*N* = 2900–3200; Hammill et al. 2023), and the St. Lawrence Estuary (*N* = 1850; DFO 2023). These four DUs are either considered Endangered or Threatened by COSEWIC and, except for the St. Lawrence Estuary DU, are still harvested for subsistence by Inuit communities in their summer aggregations or during their seasonal migrations. The low abundance of these DUs contrasts with those from the Eastern High Arctic‐Baffin Bay (*N* = 12,000, Watt et al. 2023), Western Hudson Bay (*N* = 54,500, Matthews et al. 2017), and James Bay (*N* = 19,200; DFO 2022), which were either historically exploited to a lesser extent or are recovering in numbers.

Beluga whales undertake spring and fall migrations. Mating is believed to occur when whales reside in their wintering areas or during the spring migration when eastern Canada DUs may overlap (March to June; Citta et al. 2017; Kelley et al. 2015; Manitzas Hill et al. 2024; Richard 2014). Births occur between late spring and summer when beluga whales are either moving toward or already occupying their summer ranges. During summer, beluga whales are found in shallow coastal waters as well as in deep offshore waters. They also enter estuaries and sometimes move upstream into rivers (Richard 2014). Seasonal migrations can vary in extent. In eastern Canada, the Cumberland Sound, James Bay, and St. Lawrence Estuary DUs move short seasonal distances on the scale of a few tens to hundreds of kilometers, whereas the Eastern High Arctic‐Baffin Bay, Western Hudson Bay, and Eastern Hudson Bay DUs migrate seasonally over several hundreds or thousands of kilometers (Bailleul et al. 2012a; Lewis et al. 2009; Luque and Ferguson 2010; Richard 2014). Recent evidence suggests beluga whales travel along the same seasonal migration routes each year, likely learned through strong mother‐calf bonding (Colbeck et al. 2013; Krasnova et al. 2014; O'Corry‐Crowe et al. 2018).

Across their Canadian range, beluga whales were initially divided into DUs based on the strong philopatry to summering grounds. This strong intra‐ and inter‐annual site fidelity displayed by individual whales is informed by lines of evidence based on studies of behavior (Caron and Smith 1990), telemetry (Bailleul et al. 2012a), isotopic and trace elements (Rioux et al. 2012), as well as genetics (Brown Gladden, Ferguson, and Clayton 1997; Brown Gladden et al. 1999; Colbeck et al. 2013; De March and Postma 2003; Parent et al. 2023; Postma et al. 2012; Turgeon et al. 2012). Early studies using a short haplotype of the mitochondrial DNA (mtDNA) control region (ca. 234 nucleotides) identified that beluga whales of the Eastern High Arctic‐Baffin Bay, Eastern Hudson Bay, Western Hudson Bay, and St. Lawrence Estuary DUs had distinct haplotype compositions (Brennin et al. 1997; Brown Gladden, Ferguson, and Clayton 1997; De March, Maiers, and Friesen 2002; De March, Stern, and Innes 2004; De March and Postma 2003; O'Corry‐Crowe et al. 1997). Later studies with a longer haplotype (ca. 609 nucleotides) showed the genetic distinctiveness of additional summer aggregations from Cumberland Sound, James Bay, and Belcher Islands (Parent et al. 2023; Postma et al. 2012; Turgeon et al. 2009). Whole mitogenome clades also showed some geographic specificity in eastern Canada (Skovrind et al. 2021).

While the study of mtDNA allowed to identify geographic patterns in maternally inherited loci, the study of biparentally inherited nuclear DNA (nDNA) would also be useful to identify populations, defined here as distinct groups of individuals where mating can occur between members within each group (Waples and Gaggiotti 2006). The genetic differentiation for Eastern High Arctic‐Baffin Bay, Cumberland Sound, James Bay, and St. Lawrence Estuary populations using mtDNA was corroborated by nuclear microsatellite loci (De March, Maiers, and Friesen 2002; Postma et al. 2012; Turgeon et al. 2012). No nDNA genetic differentiation was detected between the beluga whales from Eastern Hudson Bay and Western Hudson Bay, which supported the hypothesis that matrilinear lineages from these summer aggregations interbreed (Brown Gladden et al. 1999; Turgeon et al. 2012). However, these earlier genetic studies using about 10 microsatellite loci had limited power to characterize breeding populations compared to genomic approaches using thousands of single nucleotide polymorphisms (SNPs). The latter approaches using massive parallel sequencing generally enhance the detection of population structure, immigration and dispersal rates, interbreeding, and inbreeding (Allendorf et al. 2022; Funk et al. 2012; Hohenlohe, Funk, and Rajora 2021; McMahon, Teeling, and Höglund 2014; Shafer et al. 2015).

Two broad approaches are frequently used to characterize population genomic structure, namely, reduced representation and whole‐genome sequencing. Reduced representation sequencing is widely adopted to genotype many individuals from non‐model organisms, oftentimes without the aid of a reference genome (Andrews et al. 2016; Peterson et al. 2012), or for species with large genomes to reduce sequencing costs. This approach uses randomly selected short segments of the nuclear genome close to restriction sites, generally referred to as restriction‐site associated DNA sequencing (Andrews et al. 2016). Alternatively, whole genome sequencing is increasing in popularity due to the lowering cost of massive parallel sequencing and the much greater proportion of the genome covered, enabling an improved resolution of neutral and adaptive processes affecting the species (e.g., Fuentes‐Pardo and Ruzzante 2017; Lou et al. 2021). These two approaches have been successfully applied in multiple marine species of high conservation priority, such as pygmy blue whales *Balaenoptera musculus brevicauda* (Attard et al. 2018) and the vaquita *Phocoena sinus* (Morin et al. 2021). A comparison of results and possible interpretation obtained using the two approaches has seldom been conducted and would be useful to guide future studies of population structure of marine mammals, as whole genome sequencing is still expensive for species with large genomes.

The main objective of this study was to characterize the population genomic structure of beluga whales in eastern Canada using both reduced representation and whole‐genome sequencing approaches. Temporal and geographic information was coupled with the genomic datasets prepared for this study to (i) identify an association between summer location and populations, (ii) characterize populations' seasonal migratory routes, and (iii) estimate dispersal, interbreeding, and inbreeding for each population. We then discuss conservation implications in light of these results.

## 2 Methods

### 2.1 Data Collection, DNA Extraction, and Sequencing

**FIGURE 1:**
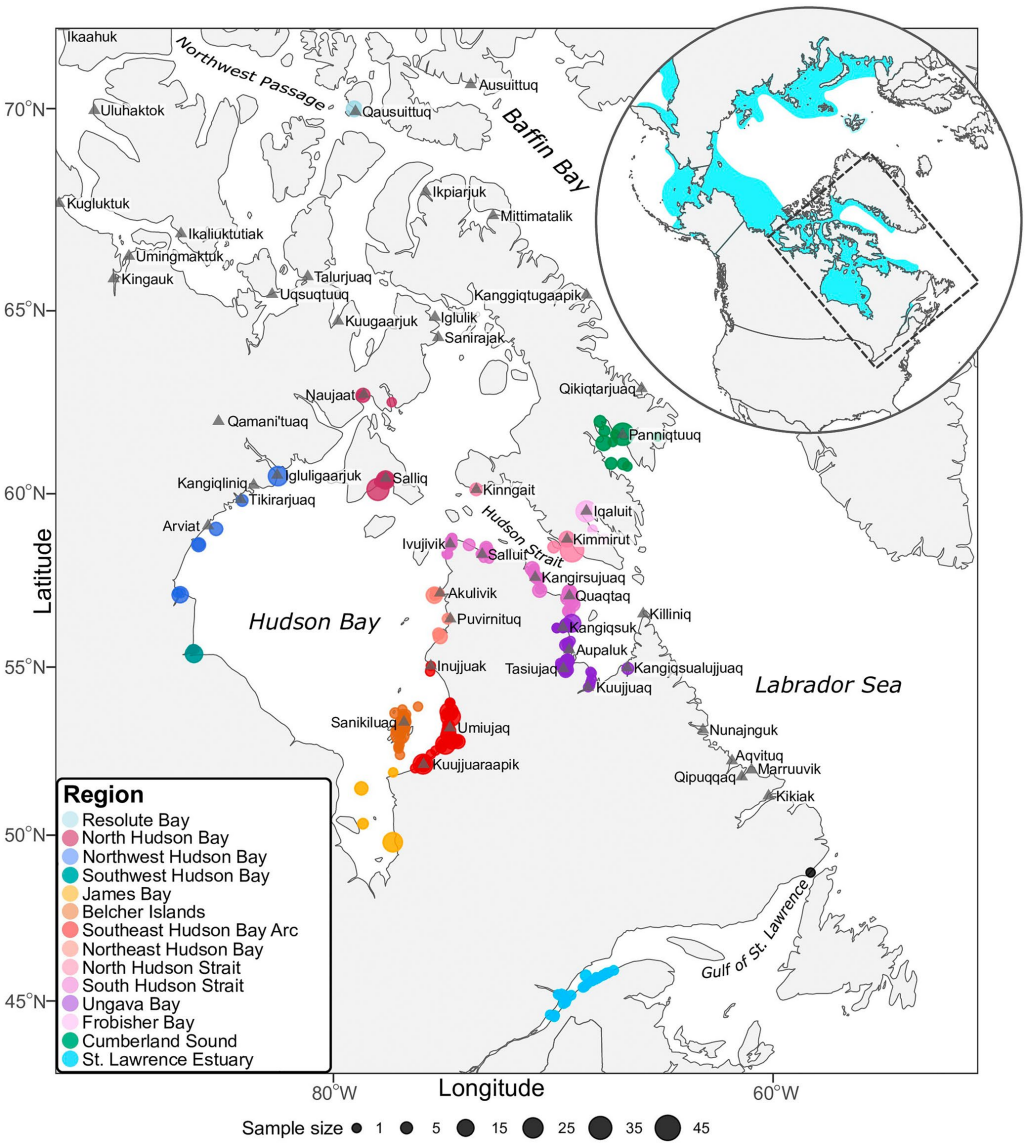
Distribution of eastern Canada beluga whales (1989–2021) genotyped using ddRADseq (*N* = 638) and lcWGS (*N* = 340) approaches. The circular insert depicts the global range of beluga whales (adapted from Hobbs et al. 2019), and a dashed rectangle outlining the study area. Only lcWGS data was produced for Resolute Bay individuals (see Table 1). A single individual is plotted in the Gulf of St. Lawrence (black dot), which is not assigned to a region.

Most whale tissue samples were provided by Inuit harvesters in the eastern Canadian Arctic as part of community‐based sampling programs during subsistence harvest. Sample metadata included harvest location (GPS coordinates, local place name, or harvester's community) and date sampled. A small subset of samples was also collected through biopsies or necropsies (Table S1). In total, 1016 samples from 14 eastern Canadian regions were collected (Table 1, Figure 1). When considering seasonal patterns, we used the following delineations, in accordance with beluga whale seasonal migratory behavior in the Arctic: winter (December–February), spring (March–June), summer (July–August), and fall (September–November; Lewis et al. 2009).

**TABLE 1:** Summary of eastern Canada samples genotyped with ddRADseq and lcWGS datasets. *N* and years indicate sample size and sampling period, respectively. *N*_overlap_ indicates the number of samples common to both datasets.

Regions^a^	ddRADseq		lcWGS		*N*_overlap_
	*N*	Years	*N*	Years	
St. Lawrence Estuary	22	2000–2019	19	2009–2019	8
Cumberland Sound	27	2002–2007	34	1982–2016	13
Frobisher Bay	16	1991–1992	8	1993–2002	0
Ungava Bay	89	1994–2018	3	2019	0
North Hudson Strait	27	1989–2000	18	1989–2009	0
South Hudson Strait	112	1994–2015	43	1994–2020	5
Northeast Hudson Bay	32	1998–2018	1	2003	1
Southeast Hudson Bay Arc	124	1990–2018	29	1990–2020	5
Belcher Islands	43	2002–2005	88	1993–2021	6
James Bay	24	2002–2009	14	2002–2010	13
Southwest Hudson Bay	14	2002–2005	1	1992–2015	1
Northwest Hudson Bay	68	1992–2015	41	1993–2009	13
North Hudson Bay	39	1993–2006	31	1996–2012	7
Resolute Bay	—	—	9	1996–2012	0
Total^b^	637	1989–2019	339	1989–2021	72

^a^ One beluga whale was not associated to any region and is not included in the table. The carcass was collected in the northeastern Gulf of St. Lawrence (see Figure 1).

^b^ The totals with the removed Gulf of St. Lawrence beluga whale are 638, 340, and 73 for ddRADseq, lcWGS, and shared individuals, respectively.

Skin, blubber, or muscle samples were preserved either frozen or in a saturated salt solution containing 20% dimethyl sulfoxide (DMSO) and 0.5 mol/L ethylene diamine tetraacetic acid (Seutin, White, and Boag 1991). DNA was extracted using DNeasy Blood and Tissue kits (Qiagen, Toronto, Canada) with slight modifications from the manufacturer's protocols (see Supplementary Information). DNA quality was evaluated on a 2% agarose gel, and DNA concentration was quantified on a Synergy LX (BioTek, Santa Clara, USA) fluorescent plate reader using PicoGreen as a fluorescent marker. Sex was genetically determined through a qPCR‐based method (Parent et al. 2024).

For the reduced representation sequencing approach, libraries of double digest restriction site‐associated DNA sequencing (ddRADseq) using *Pst*I and *Msp*I restriction enzymes were prepared by the Plateforme d'analyse génomique (IBIS, Université Laval). A total of 755 samples were indexed and pooled into ddRADseq libraries (Table S1). Ninety of these samples plus another 261 samples (*N* = 351) with high DNA quality (i.e., no signs of DNA degradation on agarose gel) were selected to prepare low‐coverage whole‐genome sequencing (lcWGS) libraries using Illumina DNA Prep and dual index by Génome Québec (Montréal, Canada, Table S1). Both ddRADseq and lcWGS libraries were sequenced on Illumina NovaSeq 6000 S4 PE 150 at Génome Québec with 10% PhiX.

### 2.2 Read Processing, SNP Calling, and Filtering

For both ddRADseq and lcWGS libraries, the overall quality of reads and the presence of adapters were assessed using FastQC 0.11.9 (Andrews 2010) and multiQC 1.10 (Ewels et al. 2016). Read processing is detailed in the Supporting Information. Briefly, raw reads were trimmed and filtered using Trimmomatic 0.39 (Bolger, Lohse, and Usadel 2014) to remove adapter sequences and low‐quality reads. ddRADseq libraries were demultiplexed using the *process_radtags* module of Stacks 2.55 (Catchen et al. 2013; Rochette, Rivera‐Colón, and Catchen 2019). Reads were mapped to a chromosomal scale reference genome for beluga whales (Bringloe and Parent 2023) using either BWA‐MEM (Li 2013; Li and Durbin 2010) for ddRADseq or bowtie2 2.4.5 (Langmead and Salzberg 2012) for lcWGS. Only samples with > 96% mapped reads were retained (Table 2).

**TABLE 2:** Filtration steps of ddRADseq and lcWGS datasets for sample and single nucleotide polymorphisms (SNPs). Each row is a filtration step targeting either samples or SNPs for selection or removal, and provides retention counts combining a given step and filtering steps in preceding rows. For the lcWGS dataset, prior to pruning for linked sites, mean SNP depth was 4.45×, while site missingness was 2.6% (Figure S12).

Filtration steps	Target	ddRADseq			lcWGS	
		SNPs	Loci	*N*	SNPs	*N*
Initial samples	—	—	—	755	—	351
Reads mapping ≥ 96%	Sample	—	—	746	71,851,401	344
Mean depth coverage ≥ 5× (post *gstacks*)	Sample	—	2,138,443	680	—	—
Located outside repetitive elements	SNP	—	—	—	41,966,793	344
Biallelic	SNP	—	—	—	41,232,252	344
Located further than 5 bp of an indel	SNP	—	—	—	40,867,199	344
Not an indel (SNPs only)	SNP	—	—	—	40,312,568	344
Minor Allele Frequency (MAF) > 1% or SNP detected in > 25% samples	SNP	136,771	88,544	680	—	—
Read depth > 15× and < 29×	SNP	106,757	69,936	680	—	—
SNP < 10% missing data and sample < 30% (ddRADseq) or < 10% (lcWGS) missing loci	SNP, sample	92,115	62,195	678	39,291,750	341
Mean read depth > 5×	Sample	92,115	62,195	677	—	—
MAF > 5%	SNP	—	—	—	3,000,827	341
Not linked to sex^a^	SNP	—	—	—	2,946,667	341
Observed heterozygosity < 60%	SNP	91,967	62,069	677	2,922,691	341
Sequencing plates effect	SNP	90,117	61,159	677	—	—
Sex‐linked and located within repetitive elements	SNP	88,433	60,102	677	—	—
Relatedness (Φ < 0.25)	Sample	88,433	60,102	638	2,922,691	341
One SNP per locus	SNP	60,102	60,102	638	—	—
MAF > 5% and < 5% missing data		26,019	26,019	638	—	—
Unlinked loci (*r* ^2^ < 0.25), 50 kbp sliding window	SNP	—	—	—	845,731	340^b^
Final dataset complete		26,019	26,019	638	845,731	340^b^
Final dataset without outliers		24,709	24,709	638		

^a^ The Y chromosome was not compiled into the original VCF file and sites on the X chromosome were removed.

^b^ One individual was removed from the final dataset due to conflicting genetic signal in replicate samples.

Aligned read information was then compiled, and variant positions were called using Stack for the ddRADseq dataset (Table 2) and a combination of SAMtools 1.12 and BCFtools 1.16 (Danecek et al. 2021) for the lcWGS (Table 2). Descriptions of SNP calling and filtration steps are detailed in Supporting Information. Briefly, for the ddRADseq, the population module of Stacks was used to first select SNPs found in at least 75% of individuals and with a minor allele frequency (MAF) of ≥ 0.01 (Table 2). For the lcWGS, only biallelic SNPs (no indels) and sites located greater than 5 bp from indels were retained (Table 2). In both ddRADseq and lcWGS datasets, sites with high missingness (> 5% and > 10% for ddRADseq and lcWGS datasets, respectively), > 60% heterozygosity, identified on the sex chromosome or located within previously identified repeat elements (Bringloe and Parent 2023) were discarded (Table 2). Individuals with high missingness (> 30% and > 15% for ddRADseq and lcWGS datasets, respectively) and with a kinship coefficient ≥ 0.25 (i.e., first‐degree relationships and putative duplicate samples; Manichaikul et al. 2010) were excluded (Table 2). For the ddRADseq dataset, SNPs were further screened to identify potential sequencing plate effects and only one SNP per locus was kept (Table 2). Finally, for the lcWGS dataset, linked sites were identified and removed with Plink 1.90 (Purcell et al. 2007), using a 50 kbp sliding window and an *r* ^2^ threshold of 0.25 (correlation coefficient of 0.5; Table 2). Final datasets for both ddRADseq and lcWGS retained SNPs with a MAF > 0.05 (Table 2; N_ddRADseq_ = 638, N_lcWGS_ = 340 individuals). Outlier loci were also identified in the ddRADseq dataset using PCAdapt 4.3.3 (Privé et al. 2020), an individual‐based genome‐scan approach that identifies SNPs significantly associated with genetic structure underlying principal components (PCs) using Mahalanobis distance. The number of PCs used was *K* = 4 based on a visual inspection of the scree plot. SNPs with a *q*‐value < 0.05 were identified as outliers (*N* = 1310). All population genomic analyses described below were run with ddRADseq SNPs including or excluding outlier loci. For the lcWGS dataset, another separate VCF file was also compiled with invariant positions included, to be used for genomic differentiation (*F*_ST_) estimates (see Supporting Information for details).

Mitochondrial genomes were assembled from the lcWGS datasets using NOVOPlasty 4.2 (Dierckxsens, Mardulyn, and Smits 2016) and cytochrome c oxidase I as a seed sequence. Mitogenomes were aligned with currently published mitogenomes (e.g., Skovrind et al. 2021) using MAUVE alignment (Darling et al. 2004) in Geneious Prime 2023 (Kearse et al. 2012).

### 2.3 Population Genomic Analyses

Samples from all seasons and regions were included in clustering analyses to identify any genomic clusters present across the study area. We evaluated the number of genetic clusters (i.e., ancestral lineages) by estimating the genetic ancestry of each individual through the model‐based ancestry estimation approach of ADMIXTURE 1.3.0 (Alexander, Novembre, and Lange 2009). The ancestry proportion (*Q*) to each K‐group (1–10 groups) was obtained for each individual for both ddRADseq and lcWGS datasets. By inspecting ADMIXTURE output, a *Q* value threshold of 0.5 was determined as a suitable threshold for assigning individuals to genomic clusters. Identifying the optimal K in admixture modeling has long been recognized as a challenging issue for model‐based ancestry estimators (Janes et al. 2017; Liu et al. 2020). The visual inspection of ADMIXTURE results was used to determine K of each dataset (Alexander, Novembre, and Lange 2009). Furthermore, the comparison of results between ADMIXTURE and the Principal Components Analysis (PCA; described below) was also used to provide insights and confirmation regarding population structure (Liu et al. 2020). For the ddRADseq dataset, PCAs were performed using the *glPca* function of the package adegenet 2.1.10 (alleles centered, missing values replaced by mean genotype across individuals; Jombart 2008; Jombart and Ahmed 2011) in R (R Core Team 2024). For the lcWGS dataset, PCAs were performed using Plink using the *–pca* flag. A PCA using adegenet was also performed on mitochondrial genomes.

*F*_ST_ estimates were used to assess the magnitude of differentiation among genomic clusters identified by the consensus of ADMIXTURE and PCA analyses. For the ddRADseq dataset, pairwise *F*_ST_ and associated 95% confidence intervals were computed from 999 bootstraps using the function *gl.fst.pop* of the R package dartR 1.0.2 (Gruber et al. 2018). For the lcWGS dataset, Pixy 1.2.7 (Korunes and Samuk 2021) was used to account for invariant positions and missing genotypes in *F*_ST_ calculations. *F*_ST_ was estimated at 50 kbp intervals, and windows with less than 15 SNPs were discarded (< 0.75% of estimates, the average number of SNPs/window was 109). The *F*_ST_ obtained with ddRADseq and lcWGS datasets were compared using Spearman's rank correlation.

Inbreeding coefficients (*F*) were estimated on a per‐individual basis from individual heterozygosity measures for both ddRADseq and lcWGS datasets using the *–het* function in VCFtools (Danecek et al. 2011). *F* was then estimated per genomic cluster as the median *F* estimates of its constituent individuals (i.e., those with *Q* ≥ 0.5 for that cluster). The Mann–Whitney *U* test was adopted to compare the distributions of *F* values among genomic clusters.

## 3 Results

A total of 905 beluga whales were included in the final SNP datasets (N_ddRADseq_ = 638 whales, N_lcWGS_ = 340 whales), with 73 specimens common to both datasets (Table 1). After SNP filtering, individuals were genotyped with 26,019 and 24,709 SNPs with ddRADseq datasets including or excluding outlier loci, respectively. Because results obtained from the two ddRADseq datasets were highly similar, results from the ddRADseq dataset including outlier loci are presented solely in the Supporting Information. A total of 845,731 SNPs were retained after filtering for beluga whale samples with the lcWGS dataset. Genotyped loci represented about 1.2% of the initial number of loci or SNPs for both datasets (Table 2). A total of 337 novel mitochondrial genomes were assembled from the lcWGS data, which were integrated with the global distribution of mitogenomes published by Skovrind et al. (2021) (Table S1, Figure S1). Sex was genetically determined for most whales (873/905, or 96.5%).

### 3.1 Main Beluga Whale Genomic Clusters in Eastern Canada

**FIGURE 2:**
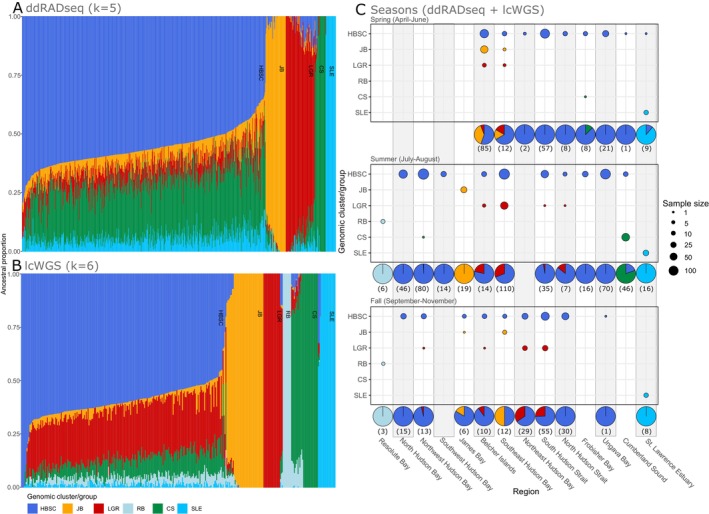
Ancestral proportions obtained with ADMIXTURE for beluga whales from eastern Canada, 1989–2021. Panels A and B represent ADMIXTURE membership probabilities for the ddRADseq (without outlier loci) and the lcWGS datasets, respectively. Colors in panels A and B represent the clusters detected. For full results at K = 2–10 sorted by geographic location, see Figures S2–S4. Panel C characterizes temporal and regional variation in the proportion of individuals from each cluster identified by ADMIXTURE, combining results from both SNP datasets (the *Population genomic analyses* section in Methods describes how individuals with mixed ancestry were classified into clusters). Sample sizes are presented below each pie chart for each region and season. Since all winter samples (*N* = 8) were harvested in the Belcher Islands region, winter results are not shown in Panel C, but seven beluga whales were associated to the JB cluster and one to the HBSC cluster. Acronyms correspond to the following genomic clusters: CS = Cumberland Sound; HBSC = Hudson Bay‐Strait Complex; JB = James Bay; LGR = Little and Great Whale Rivers; RB = Resolute Bay; SLE = St. Lawrence Estuary.

ADMIXTURE results identified several main genomic clusters of beluga whales which were consistent between the ddRADseq and lcWGS datasets (Figure 2A,B). The visual inspection of ADMIXTURE results suggested at least five clusters in common between the ddRADseq and lcWGS datasets (Figures S2–S4). The lcWGS ADMIXTURE results presented an additional cluster owing to the additional inclusion of samples from Resolute Bay, which were not included in the ddRADseq dataset (Figure 2A,B; Figure S2).

During summer, four of the five clusters identified by both datasets were strongly associated with unique summering areas, and were therefore named after these areas: the St. Lawrence Estuary (SLE), the James Bay (JB), the Little and Great Whale Rivers (LGR), and the Cumberland Sound (CS) clusters (Figure 2C). The fifth group of individuals common to both datasets had mixed ancestry proportions and was named the Hudson Bay‐Strait Complex (HBSC) cluster (Figure 2A,B). Animals in this cluster comprised the majority of the samples collected (78.5%) and were found in most regions during summer (Figure 2C). A sixth cluster, named Resolute Bay (RB), was identified only with the lcWGS dataset given none of these individuals were genotyped with ddRADseq (Figure 2B).

**FIGURE 3:**
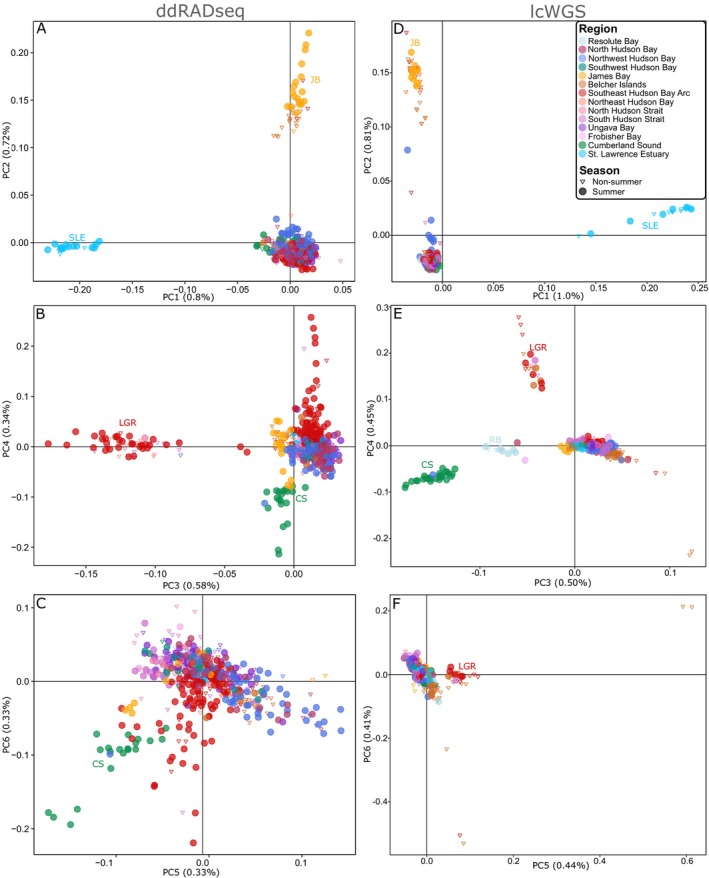
Results from the Principal Component Analyses (PCA) of eastern Canada beluga whales using ddRADseq (without outlier loci; A, B, C) and lcWGS (D, E, F) datasets. Depicted are PC axes 1–2 (panels A, D), 3–4 (panels B, E), and 5–6 (panels C, F). Figure S6 presents percent variation explained by other PC axes. See Figure S5 for the comparison of ddRADseq PCA results with outlier loci. Colors represent sampling regions (Table 1), and acronyms correspond to the following genomic clusters: CS = Cumberland Sound; JB = James Bay; LGR = Little and Great Whale Rivers; RB = Resolute Bay; SLE = St. Lawrence Estuary.

PCA results obtained with both datasets were in agreement with the ADMIXTURE results (Figure 3, Figure S5). The proportion of total variance explained by PCs decreased steeply from PC1 to PC3 with both datasets and slightly from PC4 to PC8 and from PC4 to PC5 with ddRADseq and lcWGS datasets, respectively (Figure S6). With both approaches, PCs1 and 2 showed a clear distinction between SLE and JB clusters from other beluga whales (Figure 3A,D), whereas PCs 3 and 4 revealed an additional separation of the LGR and CS clusters from other beluga clusters (Figure 3B,E). The RB cluster was separated and intermediate to the CS and the rest of the beluga whales cluster on PC3 with the lcWGS dataset (Figure 3E). Missing data and heterozygosity were not associated with clustering patterns (Figures S7 and S8).

**FIGURE 4:**
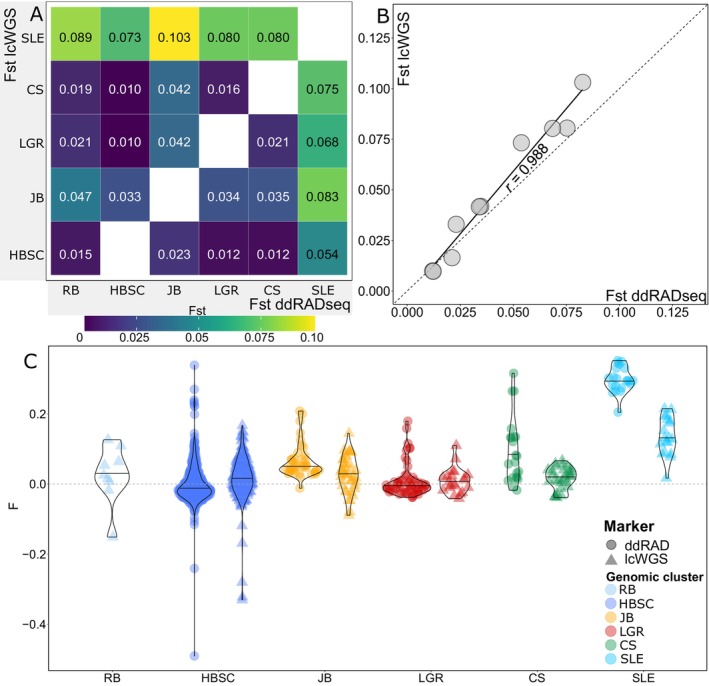
Population statistics for beluga whale genomic clusters in eastern Canada. (A) Heatmap of average *F*_ST_ measurements for ddRADseq (without outlier loci) and lcWGS datasets. (B) Correlation of *F*_ST_ estimates for genomic clusters identified with the ddRADseq (without outlier loci) and the lcWGS datasets. (C) Inbreeding coefficient (*F*) estimated with the ddRADseq (without outlier loci) and lcWGS datasets. Horizontal lines within violin distributions represent the median. Acronyms correspond to the following genomic clusters: CS = Cumberland Sound; HBSC = Hudson Bay‐Strait Complex; JB = James Bay; LGR = Little and Great Whale Rivers; RB = Resolute Bay; SLE = St. Lawrence Estuary. RB individuals were not genotyped with ddRADseq.

The *F*_ST_ between the six genomic clusters supported in the ADMIXTURE and PCA analyses varied between 0.012 and 0.083 for the ddRADseq and 0.010 and 0.103 for the lcWGS datasets (Figure 4A). With both datasets, the SLE and the JB clusters were the first and second most differentiated (Figure 4A, Figure S9). All *F*_ST_ comparisons were statistically significant for the ddRADseq dataset. The correlation between *F*_ST_ values estimated with ddRADseq and lcWGS datasets was high (rho = 0.988, *p* < 0.001; Figure 4B).

Samples associated with the JB and the HSBC clusters showed potential signs of substructure. PCA results seem to suggest two subclusters within the JB cluster as well, with samples collected in James Bay during summer or Belcher Islands during winter not completely overlapping along the PC2 axis with the ddRADseq dataset (Figure 3A). Similarly, ancestry proportions within the HBSC cluster pointed to geographically distinct patterns. Geographic variation in ancestry proportions was present at K ≥ 6 for the ddRADseq and K ≥ 7 for the lcWGS datasets (Figures S2 and S3). Results from both ddRADseq and lcWGS presented a change in ancestral composition between the Belcher Islands and Southeast Hudson Bay Arc (Figures S2, S3, S11). With the PCA, samples from some regions such as North Hudson Strait, South Hudson Strait, and Ungava Bay did not completely overlap with those from Western Hudson Bay or Southeast Hudson Bay Arc along PCs 5 and 6 with both datasets (Figure 3C,F).

### 3.2 Seasonal Migratory Patterns of Main Genomic Clusters in Eastern Canada

**FIGURE 5:**
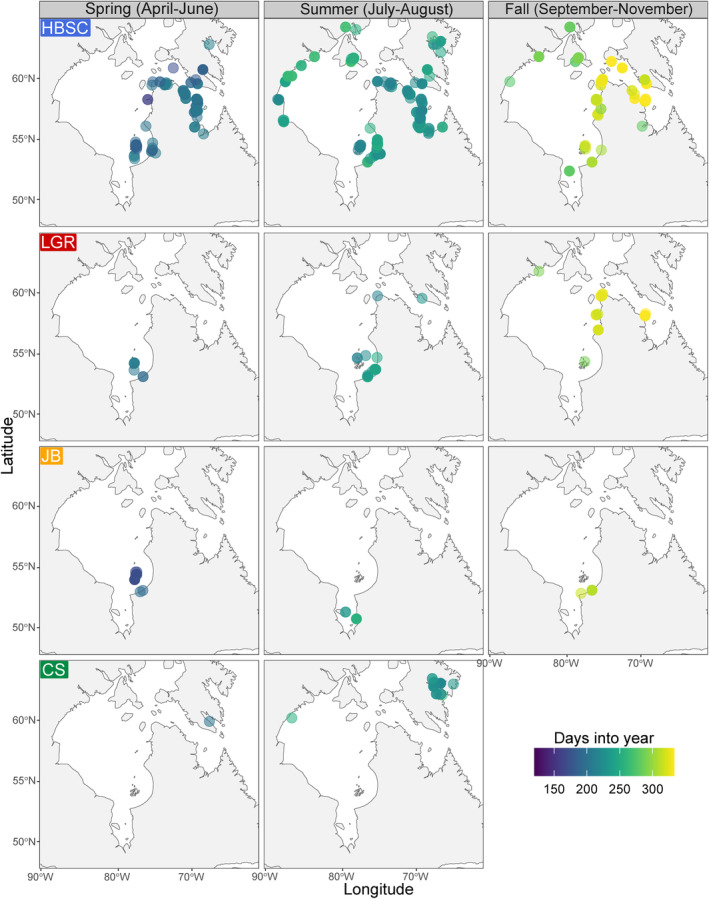
Seasonal distributions of four out of six beluga whale genomic clusters (Hudson Bay‐Strait Complex—HBSC, Little and Great Whale Rivers—LGR, James Bay—JB, and Cumberland Sound—CS) identified with ddRADseq and lcWGS ADMIXTURE analyses in eastern Canada, 1989–2021. The color palette provides a finer temporal resolution of sampling day within seasons across the study area. The RB and SLE clusters are not presented since these beluga whales were not detected migrating outside their summering regions. Location of winter samples (*N* = 8) is also not displayed, but all originated from the Belcher Islands.

Both sequencing approaches showed that during summer JB, RB, and SLE individuals were not found in regions other than those corresponding to their summer aggregation areas. Moreover, the RB and SLE clusters were not detected outside the Resolute Bay or the St. Lawrence Estuary, respectively, in other seasons. In contrast, JB beluga whales were harvested in spring outside of James Bay (*N* = 35), indicating a small‐scale migration. In spring, most JB beluga were harvested in the Belcher Islands (i.e., 33/35 or 92.3% of JB whales, F:M ratio of 4:28—one animal with sex unknown) and a few in Southeast Hudson Bay Arc (i.e., 2/35 whales, 0:2 F:M ratio; Figures 2C and 5). The JB cluster was also harvested outside of James Bay in the fall in Long Island (1/7 JB whales) and the Southeast Hudson Bay Arc (6/7 JB whales, 3:3 F:M ratio). All seven JB beluga samples collected in winter originated from the Belcher Islands (F:M ratio of 2:5, Figures 2C and 5).

During summer, the CS, LGR, and HBSC whales were harvested in multiple regions (Figures 2C, 5). CS whales were almost exclusively collected in Cumberland Sound (37/38 or 97.3% of CS whales; 16:21 F:M ratio), except for a single male harvested in Western Hudson Bay (2.5% or 1/38 whales; Figures 2C and 5). In spring, the only CS animal sampled was a female detected in Frobisher Bay. No CS whales were harvested during the fall. In summer, most of the LGR whales (34/39; 89.7%; 15:18 F:M ratio—one animal with sex unknown) were sampled in the Southeast Hudson Bay Arc region, mainly in and around the Little and Great Whale Rivers (30/34 whales, or 76.9%). Additionally, three LGR males (or 7.7%) were detected around the Belcher Islands as well as one male in the South Hudson Strait (2.5%) and one female in North Hudson Strait (2.5%, Figures 2C and 5). In spring, two of the seven LGR whales (28.6%; 2:0 F:M ratio) were harvested in the Southeast Hudson Bay and five in the Belcher Islands (71.4%; 1:4 F:M ratio). In fall, LGR whales were not sampled in the Southeast Hudson Bay Arc region, but mostly along the northern coast of Nunavik (Northeast Hudson Bay, South Hudson Strait; Figures 2C and 5). In early fall (September), one LGR male was harvested around the Belcher Islands, and another one in Northwestern Hudson Bay. Later in the season (October–November), 26 LGR whales were harvested, of which 10 (or 38.5%; 6:4 F:M ratio) were harvested in Northeast Hudson Bay and 14 (or 53.9%, 10:1 F:M ratio—one animal with sex unknown) in South Hudson Strait (Figures 2C and 5). The winter harvest from the Belcher Islands did not include any LGR whale, although the sample size was small. HBSC whales were sampled in almost every region during summer except Resolute Bay, James Bay, and the St. Lawrence Estuary. Nine of the 362 HBSC whales (2.5%) were sampled in Cumberland Sound during summer and the majority were males (1:8 F:M ratio; Figures 2C and 5). In spring, HBSC whales were harvested in Ungava Bay, South and North Hudson Strait, and Eastern Hudson Bay (Northeast Hudson Bay, Southeast Hudson Bay Arc, Belcher Islands). Spring samples from Western Hudson Bay (Southwest Hudson Bay, Northwest Hudson Bay, North Hudson Bay) were not available for this study. HBSC fall samples originated from individuals harvested from almost all Hudson Bay and Strait regions (except Southwest Hudson Bay), including northern James Bay (Figures 2C and 5). Similar to the LGR cluster, the temporal distribution of HBSC whales during fall suggests a seasonal movement toward the Hudson Strait at that time of year (September to November; Figures 2C and 5). HBSC whales were harvested in the Hudson Bay in early fall (September) and later in the Hudson Strait (October–November; Figure 5).

### 3.3 Inbreeding, Dispersal, and Admixture of Beluga Whale Genomic Clusters

On average, the inbreeding coefficients (*F*) were close to 0 for all clusters except for SLE, which had a median value ± SD of 0.255 ± 0.036 with the ddRADseq and 0.131 ± 0.053 with the lcWGS datasets (Figure 4C, Table S2). Statistically different *F* estimates were detected between all clusters with the ddRADseq dataset, except for the LGR vs. HSBC and JB vs. CS clusters (Table S2). In contrast, statistically different *F* estimates were detected only for the SLE cluster versus the JB, LGR, CS, and HSBC clusters with the lcWGS dataset (Table S2). Observed vs. expected site heterozygosity notably departed from 1:1 for lcWGS, with observed heterozygosity being underestimated at higher values of expected heterozygosity (Figure S10).

The strong association between summering areas and genomic clusters presented in the previous sections provides insight as to whether temporary or permanent dispersal is common among summering aggregations of beluga whales in eastern Canada. Individuals detected in a summering area used prominently by whales of a different cluster were identified as dispersing whales (Table S1, Figures 2C and 5). For the St. Lawrence Estuary and Resolute Bay regions, all seasons were considered when identifying dispersing whales due to the spatial disjunction with other regions. Seventeen dispersing whales were identified out of 500 animals (3.2%) using both ddRADseq and lcWGS datasets (Figures 2C and 5). Dispersing beluga whales belonged to the HBSC (*N* = 11 out of 363 whales, or 3.0%), CS (*N* = 1 out of 38 whales, or 2.6%), and LGR (*N* = 5 out of 39 whales, or 12.8%) clusters (Figure 2C). More than half of these dispersers were identified in Cumberland Sound (*N* = 9 HBSC whales), while the rest were detected in South and North Hudson Strait (*N* = 2 LGR whales), Belcher Islands (*N* = 3 LGR whales), Northwest Hudson Bay (*N* = 1 CS whale), and the St. Lawrence Estuary (*N* = 1 HBSC whale sampled inside the summer range of SLE whales; Figures 2C and 5). An additional HBSC female carcass was sampled in the eastern Gulf of St. Lawrence, that is, outside the summer range of SLE whales, indicating the possible path from the northern populations to the isolated SLE population (Figure 1). The sex ratio of the 17 dispersers was male‐biased (13 males, 4 females; Table S1).

Interbreeding between genomic clusters was identified by inspecting the individual ancestry proportions obtained with ADMIXTURE. Only the lcWGS dataset was used given the higher resolution (i.e., precision) and greater number of clusters detected. Detecting interbreeding within the HBSC cluster was impractical because of its mixed ancestry, thus interbreeding was estimated solely for beluga whales from the SLE, JB, LGR, CS, and RB clusters (*N* = 118). A total of 27 out of 118 beluga whales (23%) had mixed ancestry proportions, suggesting successful interbreeding between genomic clusters (Figure 2B). Two admixed SLE individuals with ancestry proportions close to half of the other clusters (Ancestry Proportion to Other Clusters or APOC: 0.41 and 0.43) were observed and represented about 10% (2/19 whales) of the SLE individuals (Figure 2B). Evidence of past interbreeding between clusters was present in almost half of CS whales (13/29 samples, APOC: 0.131–0.325), 35% of JB whales (14/40 samples, APOC: 0.012–0.276), and 14% for LGR whales (3/21 samples, APOC: 0.280–0.336; Figure 2B). The profile of ancestry proportions differed between the admixed beluga whales from SLE, CS, and other clusters. The two admixed SLE whales had no ancestry proportion from the RB cluster, whereas all admixed CS whales had a larger ancestry proportion of the RB cluster compared to all other admixed beluga whales (Figure 2B). Most admixed beluga whales from JB and LGR had ancestry proportions from all 4 other clusters, that is, SLE, CS, RB, HBSC (Figure 2B).

### 3.4 Association Between Mitogenome Clades and Genomic nDNA Clusters

The association between mitogenome clades and nDNA clusters was incomplete (Table S1). While geographical patterns were present, all three clades were identified in multiple regions during summer (Figure S1). The most frequent mitogenome clade was D, with 68% of whales (220/324) possessing clade D haplotypes, while 18% and 14% of whales had haplotypes from clades A and C, respectively, in our lcWGS dataset (Table S1). Clade D haplotypes were identified in all clusters but were strongly associated with the HBSC (185/216 or 86% whales), CS (19/25 or 76% whales), and RB (7/9 or 78% whales) clusters. Clade A haplotypes were most frequent in beluga whales of the SLE (15/19 or 79%) and LGR (16/21 or 76%) clusters. They also represented a minor proportion of the JB (9/34 or 27%) and HBSC (19/216 or 9%) clusters. Clade C haplotypes dominated among JB cluster whales (22/34 or 65% whales) but were also identified in the SLE, CS, RB, and HBSC clusters (Figure S1; Table S1).

## 4 Discussion

Six genomic clusters were identified by applying reduced representation (ddRADseq) and low‐coverage whole genome sequencing (lcWGS) to genotype 905 beluga whales from eastern Canada. While dispersal and interbreeding were observed between summer aggregations, these phenomena seemed relatively rare and insufficient to weaken the integrity of the genomic clusters. Moreover, both genotyping approaches were consistent in indicating potential subclusters within the HBSC and JB clusters. In the following sections, we first compare our results on population structure with those obtained in other studies and put these in the perspective of metrics available from the literature for other species including cetaceans. In the last two sections, we discuss the implications of our results for the conservation of beluga whales' diversity both in Canada and worldwide.

### 4.1 Beluga Whales Summer Aggregations Represent at Least Six Distinct Populations in Eastern Canada

Previous studies have reported isolated populations that match beluga whale summer aggregations based on mtDNA and microsatellite loci, suggesting maternal fidelity and natal philopatry toward summering areas (Brown Gladden, Ferguson, and Clayton 1997; De March, Maiers, and Friesen 2002; O'Corry‐Crowe et al. 1997, 2018; Postma et al. 2012; Turgeon et al. 2012). Philopatry to summer aggregations has been reported by behavioral studies (Bonnell et al. 2022; Caron and Smith 1990) and is confirmed again here with complete mitochondrial genomes (Figure S1). Natal philopatry, however, was weakly associated to reproductive isolation investigated in past studies using nDNA. The weak differentiation described using microsatellite loci for the Eastern High Arctic‐Baffin Bay, Cumberland Sound, James Bay, and St. Lawrence Estuary summer aggregations suggested the presence of reproductively isolated populations, while not distinguishing the summer aggregations in Southeast Hudson Bay Arc and Western Hudson Bay (De March, Maiers, and Friesen 2002; Postma et al. 2012; Turgeon et al. 2012). Our study clearly provided support for the reproductive isolation of these four genomic clusters and also revealed a newly identified, highly geographically restricted genomic cluster of whales summering in two rivers of the Southeast Hudson Bay Arc region, that is, LGR.

To a certain extent, our results are not surprising considering that nuclear genomic datasets offer insights into fine‐scale population structure missed by lower‐resolution nuclear loci (Allendorf et al. 2022; Hohenlohe, Funk, and Rajora 2021). The increased number of nuclear loci used to genotype beluga whales provided clear‐cut differences between clusters and clearly improved our understanding of reproductive isolation between summer aggregations. Similarly, upscaling resolution from microsatellites to reduced representation SNP datasets identified a greater number of populations among Arctic charr *Salvelinus alpius* (Layton et al. 2020), pike *Exos lucius* (Sunde et al. 2020), brown trout *Salmo trutta* (Lemopoulos et al. 2019), and harbor porpoise *Phocoena phocoena* (Lah et al. 2016). Our results were surprising in the detection of a genomic cluster, that is, LGR, inhabiting mainly two estuaries and migrating long distance seasonally to possibly overwinter in a common area with other groups. Such a finding suggests that population genomic studies of polar marine mammals should maximize the spread of samples throughout the entire summer distribution to identify geographically highly restricted populations. Processes underlying genomic divergence among small groups of cetaceans have been identified in other whales (Whitehead, 2017). In killer whales, for example, the colonization of novel habitats was facilitated by behavioral adaptation to various ecological niches (Foote et al. 2016). Such a hypothesis is less applicable to beluga whales, which are considered opportunistic feeders and thus less specialized. Still, there could be other behavioral processes promoting genome‐culture evolution and genomics divergence patterns among beluga whales in eastern Canada.

The levels of genomic differentiation observed among the six beluga whale clusters were similar to those that have been reported for relatively isolated terrestrial mammalian populations (Jensen et al. 2020; Schweizer et al. 2016; Yang et al. 2016). While the nearly ubiquitous HBSC cluster was the least differentiated, estimates of *F*_ST_ for other clusters ranged from 0.016 between LGR and CS to 0.103 between SLE and JB, highlighting the distinctiveness of five out of six clusters and their potential reproductive isolation. Recent studies on cetaceans using genome‐wide SNPs showed intraspecific population structure in two beaked whale species with *F*_ST_ ranging between ca. 0.020 and 0.200 (Onoufriou et al. 2022). In common dolphin *Delphinus delphis* , a complex hierarchical metapopulation structure was identified in Australia, Tasmania, and New Zealand, with isolation by distance driving genomic distances among subpopulations and *F*_ST_ ranging between ca 0.010 and 0.200 (Barceló et al. 2021). In bottlenose dolphins *Tursiops truncatus* , ecotypic differentiation drives mid‐range *F*_ST_ values of ca. 0.050–0.150 (Ansmann et al. 2012; Louis et al. 2014). Similarly, human populations feature *F*_ST_ of 0.010 within continents and 0.120 at the inter‐continental level (Elhaik 2012). These findings indicate that the level of differentiation observed among eastern Canadian beluga clusters justifies classifying them as populations. Consequently, we regard beluga whale populations and clusters as synonymous from this point onward. We believe that this terminology is reasonable and does not underestimate the level of differentiation between beluga genomic clusters. The highest level of genomic differentiation observed between beluga populations in our study is much lower than that observed between animal species (e.g., *F*_ST_ ddRADseq = 0.61 for *Sebastes* spp., Benestan et al. 2021). A relevant example with marine mammals would be killer whale ecotypes, that is, “resident” and Bigg's, which were recently recognized as distinct species. These species also exhibited genomic differentiation (*F*_ST_ genome = 0.32, Morin et al. 2024) higher than that of beluga whale populations.

Only a few dispersing beluga whales originating from three populations were detected (*N* = 17), showing little mixing of populations during summer. These results concur with those from previous studies where limited dispersal between populations was observed in highly mobile marine species (Hoelzel 1998; Morin et al. 2021), emphasizing the strong philopatry and summer site fidelity of beluga whale populations (Brown Gladden, Ferguson, and Clayton 1997; Caron and Smith 1990; O'Corry‐Crowe et al. 1997). The sex ratio of dispersing beluga whales in our study was highly male‐biased (F:M ratio = 4:13 or 0.31), as observed in most mammals where males often disperse more frequently and further away than females (Dobson 1982; Pusey 1987). It is unlikely that this biased sex ratio among dispersers represents an artifact of male‐biased harvest (and therefore sampling) since both the sex ratio associated with the complete dataset in this study (F:M = 353:488 or 0.72) and the sex ratio of the Nunavik harvest between 1993 and 2008 (0.86; Lesage et al. 2009) are much closer to parity than the sex ratio of dispersing beluga whales described here. A similar male bias was also observed among dispersers in beluga whales from the North Pacific Ocean (74% of dispersers were adult males; O'Corry‐Crowe et al. 2018) and other cetaceans (Alexander et al. 2016; Engelhaupt et al. 2009; but see Baker et al. 2013; Kershaw et al. 2017).

The population structure results using the ddRADseq and lcWGS approaches were remarkably consistent, showing the robustness of the results (Figures 2A,B and 4B). In other studies, a gain in population structure resolution was observed from ddRADseq to lcWGS approaches for Greenland halibut *Reinhardtius hippoglossoides* (Carrier et al. 2020; Ferchaud et al. 2022; Roy et al. 2014), and Atlantic salmon *Salmo salar* (Bertolotti et al. 2020; Bradbury et al. 2015). In our study, the advantages of increased resolution to detect population structure were not obvious with the comparison of ddRADseq and lcWGS results (Figure 2A,B), possibly because ddRADseq offered sufficient resolution or that the lcWGS dataset was too small. Using ddRADseq for the study of genomic population structure in beluga whales or marine mammals with a widespread and heterogeneous distribution may be the preferred approach to efficiently allocate financial resources. Using lcWGS for species with large genomes such as beluga whales (3.3Gb) comes at a much greater cost than that of reduced representation approaches. By allocating sequencing effort across more individuals, the reduced representation approach may be more powerful at identifying small and geographically restricted populations, such as LGR. In eastern Canada, increasing the number of samples could prove critical to better describe the potential substructure suggested here for the HBSC (Figure S11)and JB populations (Figure 3A). Similarly, this approach would be valuable for all cetaceans globally, which could present locally restricted small populations presently overlooked if the genotyping costs did not allow to sample all relevant distribution areas.

### 4.2 Migratory Behavior of Beluga Whale Populations

Previous studies focused on recognized beluga whale designatable units (DUs) and used either satellite‐tagging or genetic markers (mtDNA) to describe migratory movements. The James Bay and Cumberland Sound DUs migrate only short distances within their respective regions according to these data (Bailleul et al. 2012a; Jonkel 1969; Postma et al. 2012; Turgeon et al. 2012). In contrast, the Eastern Hudson Bay and Western Hudson Bay DUs undertake long seasonal migrations between their summering areas in Hudson Bay and wintering areas in Hudson Strait and adjacent areas (Bailleul et al. 2012a, 2012b; Lewis et al. 2009; Smith 2007; Turgeon et al. 2012). Our results broadly confirmed the different migratory behaviors of these DUs and contributed some additional refinements.

For the James Bay DU, an earlier study deployed satellite transmitters on 14 beluga whales (3F:11M) and all animals remained within James Bay throughout the fall and winter until transmissions ceased (Bailleul et al. 2012a). This contrasts with the current study, where 41 out of 42 JB samples collected in spring and fall (7F:33M, 1 of unknown sex) were harvested by hunters from the Belcher Islands and from the Southeast Hudson Bay Arc, suggesting these whales do migrate into surrounding regions seasonally (Figure 5). Additionally, all JB whales harvested in winter were harvested in the Belcher Islands region (2F:14M). Differences between the two studies may reflect the limited sample size of satellite‐tagging studies relative to the large JB population, the incomplete seasonal coverage of tracking data (deployment dates in late July–August, tag failure between 5 November and 10 February; Bailleul et al. 2012a), or a possible bias introduced by tracking whales tagged in eastern James Bay, while most whales are observed in western James Bay during summer (Gosselin et al. 2002; Gosselin, Hammill, and Mosnier 2017; Kingsley 2000; Smith and Hammill 1986). Alternatively, sexual segregation might explain the strong male‐biased migratory behavior identified in our study (Loseto et al. 2006; Smith, Hammill, and Martin 1994), which could not have been captured by the tracking study since tagged animals from James Bay in Bailleul et al. (2012a) were either females or young males.

For the Cumberland Sound DU, a total of 18 whales have been equipped with satellite tags, and six provided information into the winter season indicating all animals remained at the mouth or just outside of Cumberland Sound (Richard and Stewart 2008; Watt, Orr, and Ferguson 2016). The restricted sample size from the Cumberland Sound and nearby regions in our study limits conclusions about migratory behaviors of the CS population, but the fact that only one CS whale was detected outside of Cumberland Sound (i.e., Frobisher Bay), in spring suggests this DU performs short seasonal migrations, remaining in the vicinity of its summer aggregation habitat.

The HSBC and the LGR populations were both harvested in the Hudson Bay and Strait during fall (Figure 5). Both populations summer in Hudson Bay and undertake long‐distance migrations to overwinter in the Hudson Strait, northern Ungava Bay, or possibly in the Labrador Sea (Bailleul et al. 2012a; Breton‐Honeyman et al. 2016; Lewis et al. 2009; Little et al. 2023). Satellite‐tracking studies on beluga whales summering in the western regions of Hudson Bay followed at least three different fall migratory routes. They either (i) moved up the west coast of the Hudson Bay and crossed the North Hudson Bay heading east to the Hudson Strait, or (ii) passed close to the Belcher Islands prior to reaching the northeastern coast of the Hudson Bay, or (iii) crossed the center of Hudson Bay to reach the northern Nunavik coast prior to moving toward Ungava Bay (Smith 2007). In contrast, satellite‐tagged beluga whales summering in Southeast Hudson Bay Arc have been tracked migrating along the Northeast Hudson Bay coast, often remaining within 15 ± 12 km of shore (i.e., closer to the coast than observed for whales tagged in the western regions of the Hudson Bay), to overwinter in Hudson Strait or in the Labrador Sea (Lewis et al. 2009; Bailleul et al. 2012a). Harvest location data for HBSC and LGR whales in the fall agree with findings obtained by satellite‐tagging studies, although direct migratory corridors from western regions of Hudson Bay to South Hudson Strait could not be inferred, possibly because HBSC whales have a lower risk of being harvested since they migrate farther offshore relative to whales summering in Southeast Hudson Bay Arc (Smith 2007). In spring, different migration phenologies were observed for the HBSC and LGR populations moving toward their summer aggregations in Hudson Bay. Only animals from the HSBC population were harvested in Hudson Strait and in the Northeast Hudson Bay areas, whereas the LGR population was harvested from the Southeast Hudson Bay Arc or Belcher Islands. These results suggest that LGR whales move offshore and return earlier to Hudson Bay compared to animals from the other populations.

### 4.3 Improved Understanding of Reproductive Isolation and Inbreeding Status of Eastern Canada Conservation Units

The populations identified in our study correspond to a large extent to Canadian conservation units, that is, DUs (COSEWIC 2016). The St. Lawrence Estuary, James Bay, Cumberland Sound, and Eastern High Arctic‐Baffin Bay DUs (COSEWIC 2014, 2020) correspond respectively to the SLE, JB, CS, and RB populations identified in this study. The correspondence is incomplete between genomics populations and the Eastern Hudson Bay, Western Hudson Bay, and Ungava Bay DUs, for different reasons.

The Eastern Hudson Bay DU is composed of at least two populations, that is, LGR and HBSC, based on our results. The Southeast Hudson Bay Arc and the Belcher Islands are considered the regions hosting the Eastern Hudson Bay DU, which has a Threatened status according to COSEWIC (COSEWIC 2020). The LGR is a population mainly associated with beluga whales summering in and around the Little and Great Whale Rivers, that move between the coast and the offshore Belcher Islands (Bailleul et al. 2012a; Figure 2C). The Great and Little Whale Rivers suffered from some of the most intensive whale harvests (Reeves and Mitchell 1987). It is noteworthy that the Little Whale River is nowadays the only summer aggregation area in the Southeast Hudson Bay Arc region that still hosts significant numbers of beluga whales (Sauvé et al. 2024). In contrast, the HBSC population is found in most regions of the Hudson Bay and Strait during summer and may host genomic subclusters (Figures S2 and S3). Substructure within the HBSC population and in the Eastern Hudson Bay DU was weak but apparent with both datasets (Figure 2A,B, Figures S2, S3, S11). No population specific to the Belcher Islands was identified but a change in genomic composition was observed between the animals from the Belcher Islands and Southeast Hudson Bay Arc (Figures S2, S3, S11). These results support those from Parent et al. (2023), which identified that the mtDNA lineages of beluga whales from the Belcher Islands differed from those of the Southeast Hudson Bay Arc and adjacent areas. Thus, the mtDNA and nDNA genomic information both suggests the presence of two additional populations other than LGR in the Eastern Hudson Bay DU. However, we believe that our current nDNA datasets lack the scope to provide a confident interpretation of the two subclusters. Further genotyping in this area is needed to increase certainty in identification of populations with lower genomic differentiation, a well‐known problem in population genomics analyses (Janes et al. 2017; Kalinowski, 2011; Toyama et al. 2020; Wang 2017).

The Western Hudson Bay and Ungava Bay DUs consisted only of beluga of the HBSC population. Both DUs have been identified because of their ecological disjunction from other conservation units (COSEWIC 2020; Finley et al. 1982). Our results also suggest that the genomic composition differed between beluga whales from Western Hudson Bay and Hudson Strait (Figures S2, S3, S11). Again, genotyping more animals from these two areas, especially from key areas such as Marralik River in Ungava Bay, Nelson, Churchill, and Seal Rivers in Western Hudson Bay, is necessary to clarify the substructure within the HBSC population.

Our results for inbreeding varied greatly between Threatened and Endangered DUs associated with a genomic population, that is, Eastern Hudson Bay (LGR), Cumberland Sound (CS), and St. Lawrence Estuary (SLE). Beluga whale abundances in Eastern Hudson Bay and Cumberland Sound have declined markedly due to commercial whaling. Their abundances are estimated at 2900–3200 and 1100 beluga whales, respectively, and are still declining (Hammill et al. 2023; Watt et al. 2021). Despite low population abundance, neither the LGR nor the CS populations show signs of high inbreeding coefficients (but note that 2/3 of LGR whales, and all CS whales used in this study were sampled prior to 2010; Table S1).

For the CS population, recent admixture may account for its comparable inbreeding coefficient to other large populations. Dispersers and “admixed” beluga whales were more frequent in Cumberland Sound compared to other regions, suggesting effective immigration in this area. Half of dispersing beluga whales and almost half of the admixed whales identified in this study were harvested in Cumberland Sound. Satellite tracking data of summering Cumberland Sound beluga whales suggests that these whales overwinter isolated from other populations in a persistent polynya located on the southeast side of the Sound (Richard and Stewart 2008; Watt, Orr, and Ferguson 2016). In the past, immigrants to Cumberland Sound likely overwintered and reproduced with the CS beluga whale population based on our results. Alternatively, the presence of admixed individuals, especially those with a “high” ancestral proportion belonging to the RB population, could be the outcome of interbreeding during the late winter breeding period when both populations occur in Baffin Bay (Heide‐Jørgensen et al. 2003). Despite some level of gene flow, the integrity of the CS population appears to be maintained.

In contrast, there is little evidence for extensive admixing between the LGR and the HBSC populations, discounting recent and extensive admixture as an explanation for the low inbreeding detected among LGR whales. The rare admixing events between LGR and HBSC whales are inconsistent with previous hypotheses that beluga whales from different summer aggregations in the Hudson Bay have ample opportunities to interbreed either by congregating in common wintering grounds or when migrating back to their summer areas using the same migration routes (Brown Gladden, Ferguson, and Clayton 1997; Brown Gladden et al. 1999; De March and Postma 2003). The few admixing events detected in this study support the rarity individual dispersal and admixing between populations. From a management perspective, this translates to reduced opportunities for genetic rescue of small and inbred summering aggregations, and because of the strong site fidelity highlighted in beluga whales, repopulation of summering areas due to immigration from whales of other populations is unlikely. This statement is in agreement with the Inuit Qaujimajatuqangit that report limited observations in rivers or estuaries where beluga whales used to summer (e.g., Inukjuak River, Puvirnituq Bay; Archéotec Inc. 1990).

For the St. Lawrence Estuary DU, results showed strong evidence of inbreeding despite some evidence of immigration and gene flow from eastern Arctic beluga whales. The detection of one immigrant HBSC whale in the St. Lawrence Estuary and the presence of admixed SLExHBSC individuals indicate that dispersal from the north has occurred in the past. Beluga whales were formerly much more abundant along the Labrador coast and approximately 100 years ago, an influx of beluga whales thought to be of Arctic origin was observed in the St. Lawrence Estuary, possibly in response to a change in water temperatures (Brice‐Bennett 1978; Vladykov 1944). Although beluga whale numbers in Ungava Bay and along the Labrador coast have declined, northern vagrants identified from their contaminant loads and trace element levels are still reported in the eastern Gulf of St. Lawrence and Newfoundland waters (V. Lesage, unpublished data). However, the high inbreeding coefficient observed among SLE whales indicates that these vagrants, if they venture into the SLE whales' distribution range, may not contribute in noticeable ways to the genetic diversity of this DU. Our most parsimonious hypothesis to explain the difference in inbreeding coefficients between SLE, LGR, and CS is that the SLE population underwent a strong founder effect or bottleneck prior to recent hunting and bounty programs from the 16th to the mid‐20th centuries. Demographic models using genomic data would, however, be essential to shed light on the evolution of the genetic diversity of this specific population.

### 4.4 Conclusion

The analyses in this study leveraged two methods for sampling genomic loci, demonstrating a high degree of congruency between reduced representation (ddRADseq) and whole genome (lcWGS) sequencing approaches. By analyzing a large number of individuals (> 900) and loci (ca. 24,000–846,000 SNPs), we were able to improve on the scale of genomic analyses of cetaceans, which typically feature 10–100 s of individuals and 100–1000s SNPs. We conclude that reproductively isolated units among beluga whales are consistent but not entirely synonymous with summer aggregations in eastern Canada, which currently serve as the basis for conservation management (DUs for COSEWIC; COSEWIC 2016, 2020). Mitochondrial DNA is currently used to estimate harvesting‐induced mortality across management areas (DFO 2022; Hammill et al. 2023; Parent et al. 2023). Our work, however, showed an incomplete association between clade structure identified with mtDNA and populations identified with nDNA; the impact of these results for management warrant further investigation. Also, the development of new genomic tools using SNPs to discriminate genomic clusters (e.g., GTseq; Campbell, Harmon, and Narum 2015), which are more cost‐ and time‐effective than ddRADseq and lcWGS in a monitoring program, should be a priority to quantify harvesting‐induced yearly mortality of beluga whale populations in eastern Canada. Our findings also suggest the existence of a more complex genetic structure within the HBSC population. We suspect that additional genomic clusters remain unresolved in our analysis, justifying the need to increase the scope of sampling in future analyses.

## Ethics Statement

Ethical approval was not required since tissues have been collected by hunters as part of the Nunavik and Nunavut tissue sampling program of Fisheries and Oceans Canada.

## Conflicts of Interest

The authors declare no conflicts of interests.

## Supporting information


Appendix S1.

Table S1.

## Data Availability

All raw read data were deposited with NCBI's short read archive under the BioProjectID PRJNA984210. New mitochondrial genomes are published under accessions OQ553962–OQ554323. Workflow details and scripts for compiling the lcWGS datasets are available via Github: https://github.com/tbringloe/WGS‐NOVAC. Workflow details and scripts for compiling the ddRADseq datasets and performing analyses are available at: https://doi.org/10.5281/zenodo.14004391.
