# Supplementary material for: Reduced Representation and Whole‐Genome Sequencing Approaches Highlight Beluga Whale Populations Associated to Eastern Canada Summer Aggregations
Source: Evol Appl. 2024 Dec 18;17(12):e70058. doi: 10.1111/eva.70058 (PMC11655672; doi:10.1111/eva.70058)
Supplement: Supplementary file 1 — Appendix S1. [file EVA-17-e70058-s002.docx]

# Electronic Supplementary Material for

**Reduced representation and whole-genome sequencing approaches highlight beluga whale populations associated to eastern Canada summer aggregations**

Luca Montana^a,†,^* , Trevor T. Bringloe^a,†,^*, Audrey Bourret^a^, Caroline Sauvé^a^, Arnaud Mosnier^a^, Steven H. Ferguson^b,c^, Lianne Postma^b^, Véronique Lesage^a^, Cortney A. Watt^b,c^, Mike O. Hammill^a^, and Geneviève J. Parent^a,^*

^a^ Maurice Lamontagne Institute, Fisheries and Oceans Canada, Mont-Joli, QC, G5H 3Z4, Canada

^b^ Freshwater Institute, Fisheries and Oceans Canada, Winnipeg, MB, R3T 2N6, Canada

^c^ Department of Biological Sciences, University of Manitoba, Winnipeg, MB R3T 2N2, Canada

^†^ LM and TTB contributed equally to this work

* Corresponding authors: LM ([luca.montana@usherbrooke.ca](mailto:luca.montana@usherbrooke.ca)), TTB ([tbringloe@gmail.com](mailto:tbringloe@gmail.com)), GJP ([genevieve.parent@dfo-mpo.gc.ca](mailto:genevieve.parent@dfo-mpo.gc.ca))

# Supplementary Text

## DNA extraction

DNA was extracted with either the Qiagen DNeasy Blood and Tissue kit or the Qiagen DNeasy 96 Blood and Tissue kit (Qiagen, Toronto, Canada) for QIAcube HT (Qiagen, Toronto, Canada). Manufacturer’s protocols were followed apart for tissue lysis, which occurred in doubled buffer and proteinase K volumes overnight at 56°C on an Eppendorf™ Thermomixer at 15,000 rpm. Tissues were also physically altered with a pestle after one hour of tissue digestion (Parent et al., 2024).

## Read processing, genotype calling and filtering: Double digest restriction site-associated DNA sequencing (ddRADseq)

Overall quality of reads and presence of adapters were assessed using FastQC 0.11.9 (Andrews, 2010) and multiQC 1.10 (Ewels et al., 2016). Illumina adapters as well as three base pairs (bp) from read 2 (R2) were removed from raw sequence files using Trimmomatic 0.39 (Bolger et al., 2014). The three bp removed correspond to the *MspI* restriction site, where we detected a decrease in sequence quality. Reads were then visualized again with FastQC and multiQC to ensure removal of Illumina adaptors and examine read quality anew. Reads were then processed with Stacks 2.55 modules (Catchen et al., 2013; Rochette et al., 2019). The module *process_radtags* was used for demultiplexing and quality filtering: reads were truncated to 135 bp, and *PstI* restriction site quality at read 1 (R1) was assessed. Demultiplexed reads were aligned to a chromosomal scale beluga genome assembly available on NCBI (accession ID: JARJCU000000000; Bringloe & Parent, 2023) with the Maximal Exact Match (MEM) algorithm in BWA-MEM (Li, 2013; Li & Durbin, 2010) using default parameters. To call SNPs with reference alignments, aligned reads were then sorted using SAMtools 1.12 (Danecek et al., 2021). Nine out of 755 samples with an alignment rate below 96% were discarded from further analysis (**Table 2**). Aligned pair-end reads were finally assembled with *gstacks* modules. Samples with a mean coverage below 5X (N = 66) were discarded from further analyses.

The *population* module of Stacks was used to perform the first SNPs filtration step on the 680 samples: SNPs were removed if they were not found in at least 75% of individuals, or with a minor allele frequency (MAF) of ≤ 1% (**Table 2**; median mean read depth = 16.2X). The resulting SNP panel was exported in a variant call format (VCF). This first MAF filter helps removing low-frequency SNPs miscalls (Rochette & Catchen, 2017). The second filtration step aimed to discard those loci with low read depth (or coverage). If read depth of a locus is insufficient, alleles may not be detected and thus result in false homozygotes (O’Leary et al., 2018; Rochette et al., 2019). After inspecting if some loci were more homozygous than expected by comparing observed vs expected heterozygosity, we removed loci with mean read depth ≤ 15X and with mean depth ≥ 29X with VCFtools 0.1.16 (**Table 2**; Danecek et al., 2011). These thresholds were chosen because most loci with a higher than expected homozygosity had a mean read depth between 5X and 15X, and a mean depth = 29 represents the 99^th^ percentile of the mean read depth distribution. The third filtration step aimed to discard individuals with more than 30% missing loci and loci with more than 10% missing data using VCFtools 0.1.16 (Danecek et al., 2011), which removed 1 individual and 14,642 loci (**Table 2**). Next, we removed samples that had a low mean read depth across all loci (median mean read depth across samples = 15.5X; threshold = 5X; **Table 2**), always using VCFtools 0.1.16 (Danecek et al., 2011). The fifth filtering steps used vcfR 1.13.0 (Knaus & Grünwald, 2017) to identify loci with an observed heterozygosity ≥ 60% after importing VCF files in R 4.3.0 (R Core Team, 2024). Highly heterozygous loci were discarded with VCFtools (**Table 2**; Danecek et al., 2011). Filtering for highly heterozygous loci prevents maintaining the erroneous merging of paralogous loci (Rochette & Catchen, 2017). Loci were screened to identify potential sequencing plate effects, following the procedure adapted from Capblancq et al. (2018). Briefly, a redundancy analysis (*rda* function of the R package *vegan* 2.6-2; Oksanen et al., 2022) was performed using individual genetic data as response matrix Y, and sequencing plate identifier as explanatory matrix X. The Mahalanobis distance was then computed over loci loadings of the most informative ordination axes, identified through visual observation of the scree plot (K = 3). SNPs with a *q*-value < 5.4 x 10*-7* (i.e., alpha level of 0.05 / N SNPs; N = 1,850 SNPs) were identified as outliers (**Table 2**). Sex-linked SNPs were excluded by removing loci associated to the chromosome X identified on the reference genome by Bringloe & Parent (2023), as well as SNPs located within previously identified repeat elements, were discarded with VCFtools (Danecek et al., 2011). Relatedness between samples was estimated using the method of (Manichaikul et al., 2010) available in VCFtools. High relatedness (Φ > 0.25) can be caused either because two samples belong to the same individual (Manichaikul et al., 2010), or because contamination between samples occurred. For samples with metadata indicating they belonged to the same specimen, one of the duplicates was discarded (N = 15). If contamination occurred, both samples were discarded (N = 24). This filtering step left 638 specimens for analyses (**Table 2**). We then kept one SNP per locus by selecting that with the highest read depth (**Table 2**). Finally, we re-estimated the MAF and the number of missing loci within the finalized reduced dataset (**Table 2**). We eliminated loci with more than 5% missing data and with MAF ≤ 5%, a common threshold in marine mammals studies, preventing the use of false SNPs (Rochette & Catchen, 2017).

## Read processing, genotype calling and filtering: Low coverage Whole Genome Sequencing (lcWGS)

As with the ddRADseq datasets, overall read quality and presence of adapters were assessed using FastQC 0.11.9 (Andrews, 2010) and multiQC 1.10 (Ewels et al., 2016). Using Trimmomatic 0.39 (Bolger et al., 2014), the first 15 bp were cropped from all reads (headcrop), trailing bases with quality scores of less than 20 were trimmed, Illumina adapters were screened and trimmed, and reads with an average quality score <25 or <75 bp in length were discarded. Reads were mapped to a chromosomal scale reference genome for beluga Bringloe & Parent (2023) using bowtie2 v.2.4.5 (Langmead & Salzberg, 2012) and an 85% alignment similarity threshold. Aligned read information was then compiled and variant positions were called using a combination of SAMtools 1.12 and BCFtools v. 1.16 (Danecek et al., 2021). The raw VCF file underwent the following filtration steps: samples with >96% mapped reads were retained using BCFtools; variant positions located within previously identified repeat elements Bringloe & Parent (2023) were discarded using VCFtools; biallelic SNPs (no indels) and sites located greater than 5 bp from indels were retained using BCFtools; individuals with less than 15% missingness and sites with less than 10% missingness were retained using VCFtools; the *population* module of Stacks was used to calculate observed heterozygosity at the site level, and sites with greater than 60% heterozygosity were discarded using VCFtools; sex-linked SNPs were excluded by removing SNPs identified on the X chromosome (the Y chromosome was excluded during vcf compilation); sites with a MAF <0.05 were discarded using VCFtools; individuals with a kinship coefficient greater than 0.25 (i.e., siblings) were removed; finally, linked sites were identified and removed using plink v1.90 (Purcell et al., 2007), using a 50kbp sliding window and an r^2^ threshold of 0.25 (correlation coefficient of 0.5). The full set of commands to compile and filter the vcf are available via GitHub (<https://github.com/tbringloe/WGS-NOVAC>).

A separate vcf was also compiled with invariant positions included, to be used for *F*_ST_ estimates. In order to address limitations with the vcf file size, five individuals from each of six identified genomic clusters were compiled, overlapping as much as possible with the ddRADseq datasets (22/25 samples with shared genomic clusters detected). Rather than remove SNP artifacts as above, a different approach was employed that prioritized setting genotypes to missing as needed. First, during vcf compilation, only reads with base and mapping quality scores of 30 or greater (1/1000 chance of error) were considered for calling SNPs. Following this, genotypes within 5 bp of an indel were set to missing, and we removed sites from repeat regions, indels and biallelic sites, the X chromosome, and sites with >60% heterozygosity.

Five narwhal specimens were also compiled along with the beluga specimens, generally following the same procedures as outlined above (i.e., MAF was lowered to 0.004). This dataset was analysed separately in order to ascertain whether any beluga individuals represented beluga-narwhal hybrids.

# Supplementary Tables

## **Table S1.** Metadata, mtDNA clade, and nDNA membership of 905 eastern Canada genotyped in this study.

## Table S2. Mann-Whitney U test results for departures in inbreeding coefficients among main genomic cluster for beluga whales from eastern Canada. Significant values are in bold.

| Cluster 1 | Cluster 2 | Approach | Median ± SD *F* cluster 1 | Median ± SD *F* cluster 2 | *U* | *P* |
| --- | --- | --- | --- | --- | --- | --- |
| **RB** | **SLE** | **lcWGS** | **0.025 ± 0.081** | **0.131 ± 0.053** | **16** | **< 0.001** |
| RB | CS | lcWGS | 0.025 ± 0.081 | 0.021 ± 0.031 | 155 | 0.417 |
| RB | LGR | lcWGS | 0.025 ± 0.081 | 0.006 ± 0.036 | 122 | 0.226 |
| RB | JB | lcWGS | 0.025 ± 0.081 | 0.027 ± 0.051 | 188 | 0.849 |
| RB | HBSC | lcWGS | 0.025 ± 0.081 | 0.016 ± 0.062 | 1123 | 0.432 |
| **HBSC** | **SLE** | **ddRADseq** | **-0.008 ± 0.056** | **0.255 ± 0.036** | **62** | **< 0.001** |
| **HBSC** | **SLE** | **lcWGS** | **0.016 ± 0.062** | **0.131 ± 0.053** | **233** | **< 0.001** |
| **HBSC** | **CS** | **ddRADseq** | **-0.008 ± 0.056** | **0.074 ± 0.093** | **1580** | **< 0.001** |
| HBSC | CS | lcWGS | 0.016 ± 0.062 | 0.021 ± 0.031 | 3177 | 0.901 |
| HBSC | LGR | ddRADseq | -0.008 ± 0.056 | -0.005 ± 0.046 | 12974 | 0.298 |
| HBSC | LGR | lcWGS | 0.016 ± 0.062 | 0.006 ± 0.036 | 2598 | 0.272 |
| **HBSC** | **JB** | **ddRADseq** | **-0.008 ± 0.056** | **0.043 ± 0.048** | **3334.5** | **< 0.001** |
| HBSC | JB | lcWGS | 0.016 ± 0.062 | 0.027 ± 0.051 | 3823 | 0.249 |
| **JB** | **SLE** | **ddRADseq** | **0.043 ± 0.048** | **0.255 ± 0.036** | **3** | **< 0.001** |
| **JB** | **SLE** | **lcWGS** | **0.027 ± 0.051** | **0.131 ± 0.053** | **57** | **< 0.001** |
| JB | CS | ddRADseq | 0.043 ± 0.048 | 0.074 ± 0.093 | 376 | 0.285 |
| JB | CS | lcWGS | 0.027 ± 0.051 | 0.021 ± 0.031 | 674 | 0.258 |
| **JB** | **LGR** | **ddRADseq** | **0.043 ± 0.048** | **-0.005 ± 0.046** | **2011** | **< 0.001** |
| JB | LGR | lcWGS | 0.027 ± 0.051 | 0.006 ± 0.036 | 532 | 0.091 |
| **LGR** | **SLE** | **ddRADseq** | **-0.005 ± 0.046** | **0.255 ± 0.036** | **2** | **< 0.001** |
| **LGR** | **SLE** | **lcWGS** | **0.006 ± 0.036** | **0.131 ± 0.053** | **13** | **<0.001** |
| **LGR** | **CS** | **ddRADseq** | **-0.005 ± 0.046** | **0.074 ± 0.093** | **172** | **< 0.001** |
| LGR | CS | lcWGS | 0.006 ± 0.036 | 0.021 ± 0.031 | 248 | 0.274 |
| **CS** | **SLE** | **ddRADseq** | **0.074 ± 0.093** | **0.255 ± 0.036** | **51** | **< 0.001** |
| **CS** | **SLE** | **lcWGS** | **0.021 ± 0.031** | **0.131 ± 0.053** | **15** | **< 0.001** |

# Supplementary Figures


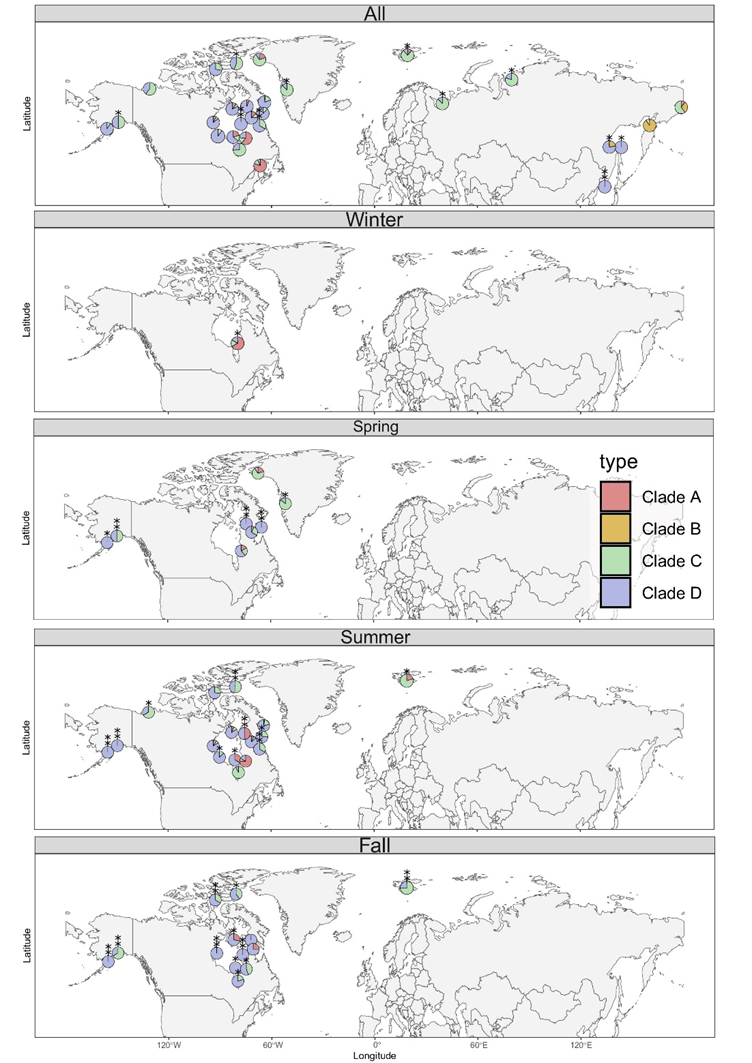


## Figure S1. Distributions of mitochondrial clades in global beluga populations. Clades follow Skovrind et al. (2021) and include publicly available genomes and newly generated mitochondrial genomes through the current study. Note, seasonal information was not available for some Russian data, which appear in the “All” panel. A single asterisk indicates locations with sample sizes <10, while two asterisks indicate samples sizes <5. Seasons are defined as: winter, December-March; spring, April-May; summer, July-August; and fall, September-November.


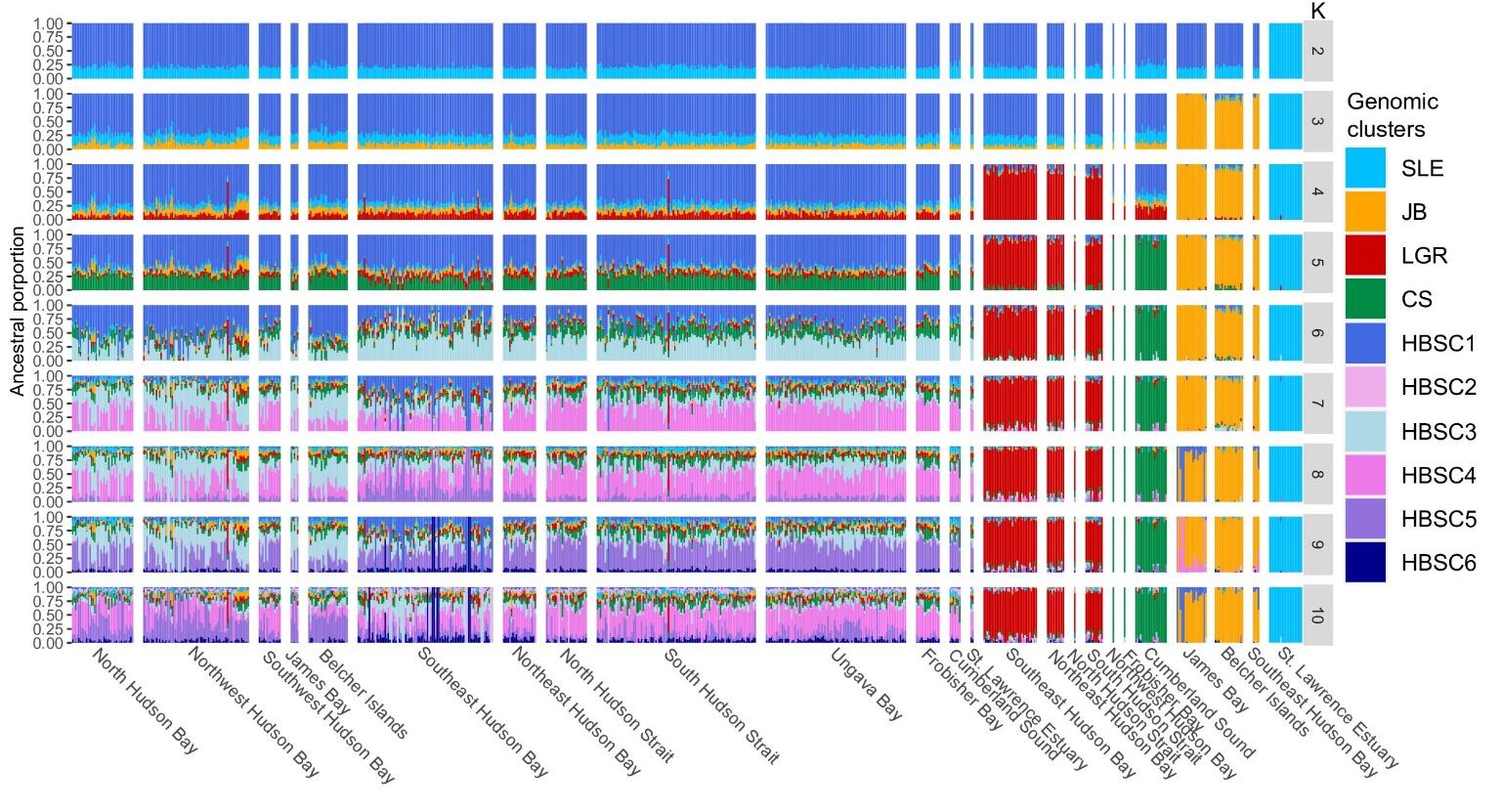


## Figure S2. ADMIXTURE results for eastern Canada beluga for K from 2 to 10 using the ddRADseq dataset without outlier loci. SLE = St. Lawrence Estuary, JB = James Bay-Belcher Islands, LGR = Little and Great Whale Rivers, CS = Cumberland Sound, HBSC = Hudson Bay-Strait Complex.


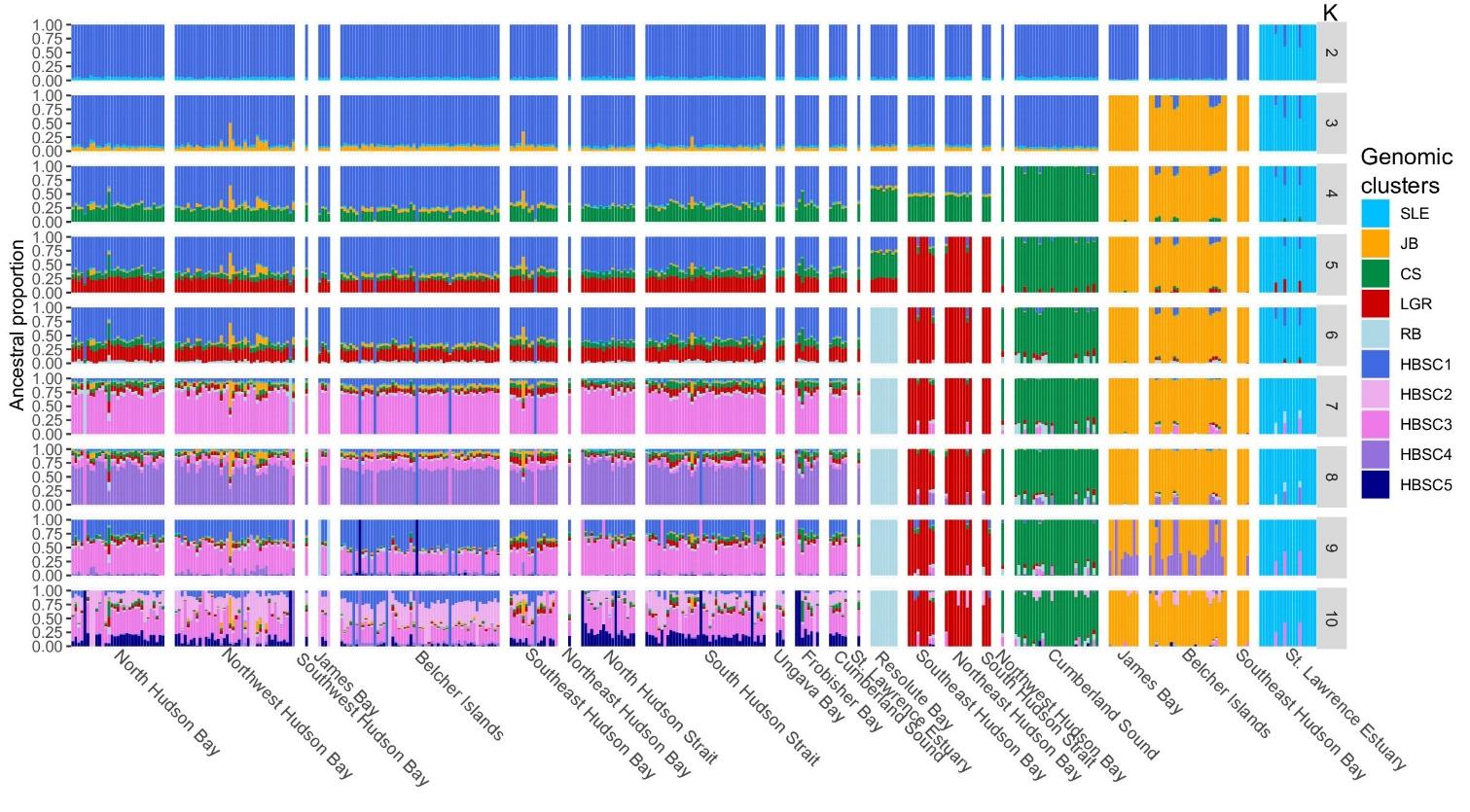


## Figure S3. ADMIXTURE results for eastern Canada beluga for K from 2 to 10 using the lcWGS dataset. SLE = St. Lawrence Estuary, JB = James Bay, LGR = Little and Great Whale Rivers, CS = Cumberland Sound, RB = Resolute Bay, HBSC = Hudson Bay-Strait Complex.


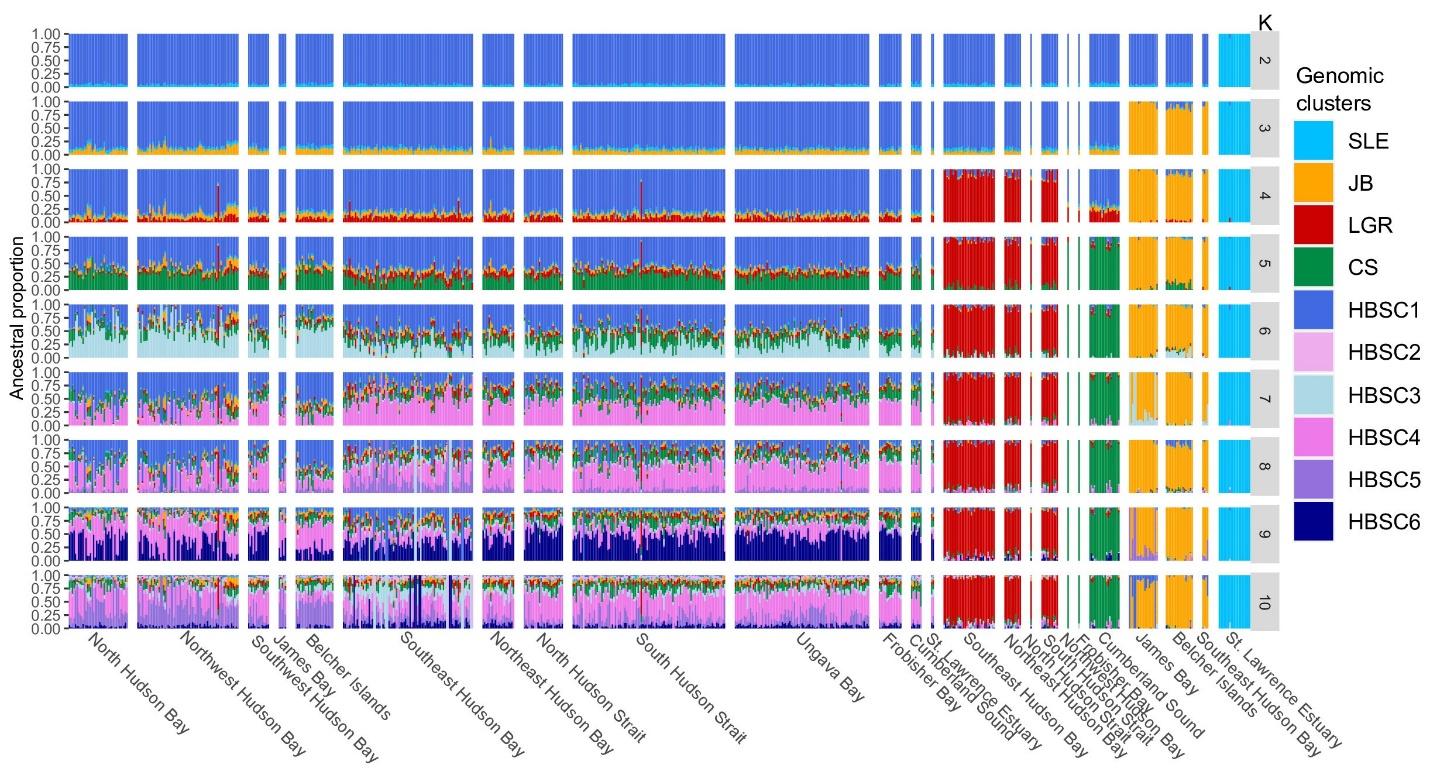


## Figure S4. ADMIXTURE results for eastern Canada beluga for K from 2 to 10 using ddRADseq dataset including outlier loci. SLE = St. Lawrence Estuary, JB = James Bay, LGR = Little and Great Whale Rivers, CS = Cumberland Sound, HBSC = Hudson Bay-Strait Complex.


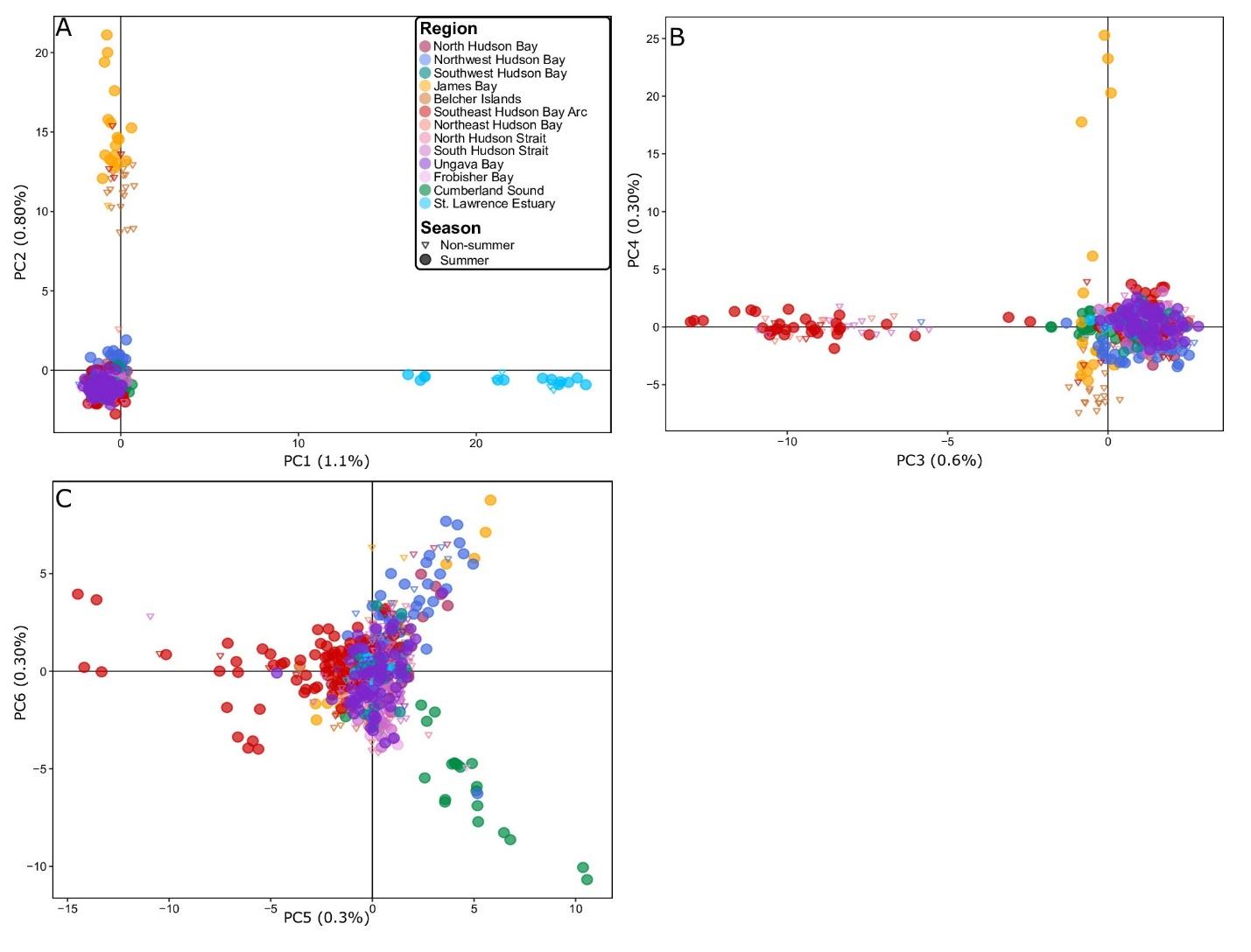


## Figure S5. Principal Component Analysis (PCA) for eastern Canada belugas using the complete ddRADseq dataset (with outlier loci) for subsets of principal components (PCs) 1 to 6.


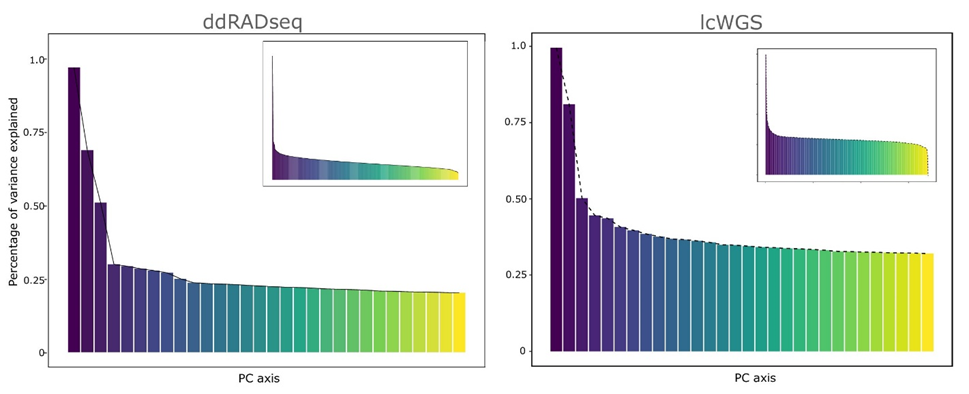


## **Figure S6.** Percentage of variation explained by the first 30 PC axes (global distribution of all PC axes in the insets) for PCAs estimated using ddRADseq and lcWGS datasets (Figure 2).


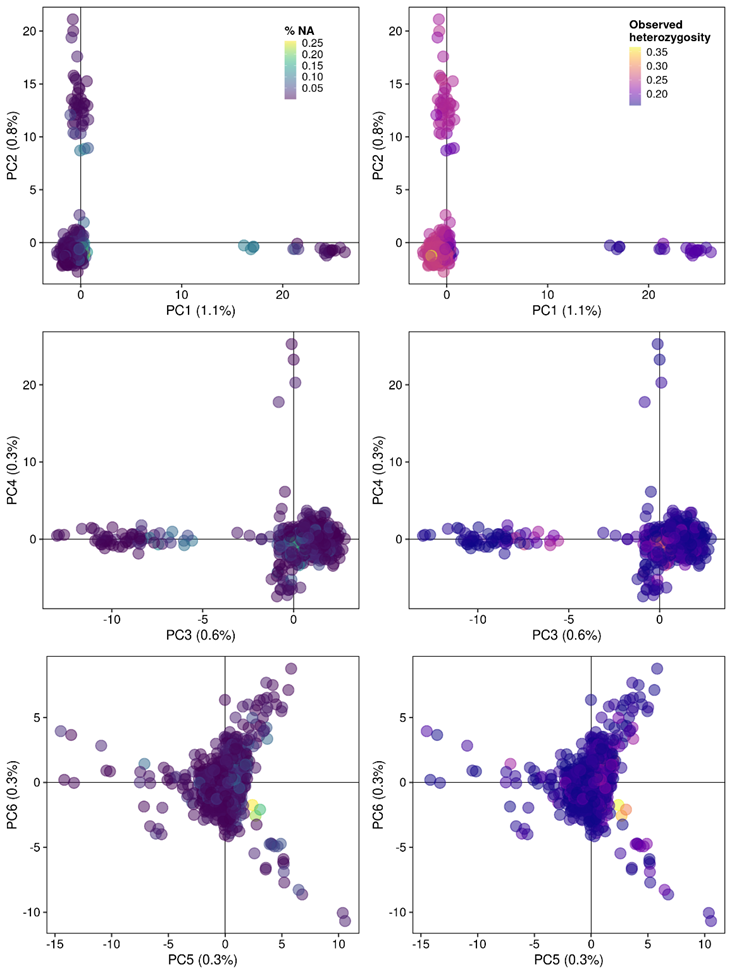


## Figure S7. Principal Component Analyses (PCAs) of eastern Canada belugas using the ddRADseq dataset and representing missing data and observed heterozygosity for subsets of principal components (PCs) 1 to 6.


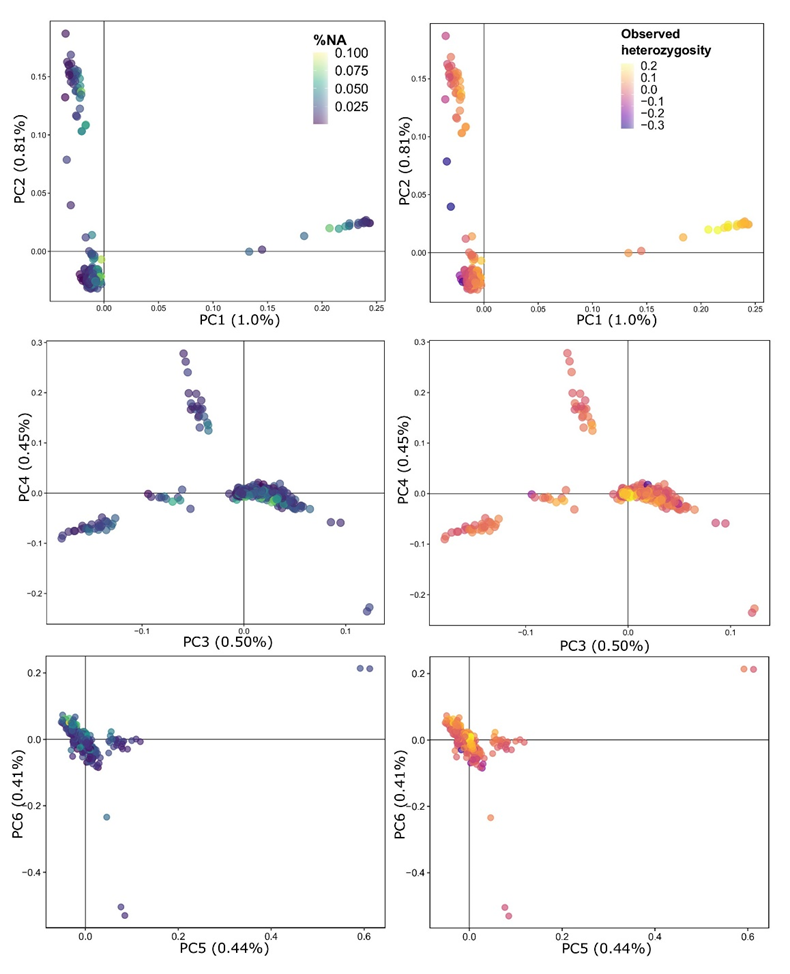


## Figure S8. Principal Component Analyses (PCA)s of eastern Canada belugas using the lcWGS dataset. Missing data and observed heterozygosity are depicted per sample for principal components (PCs) 1 to 6.


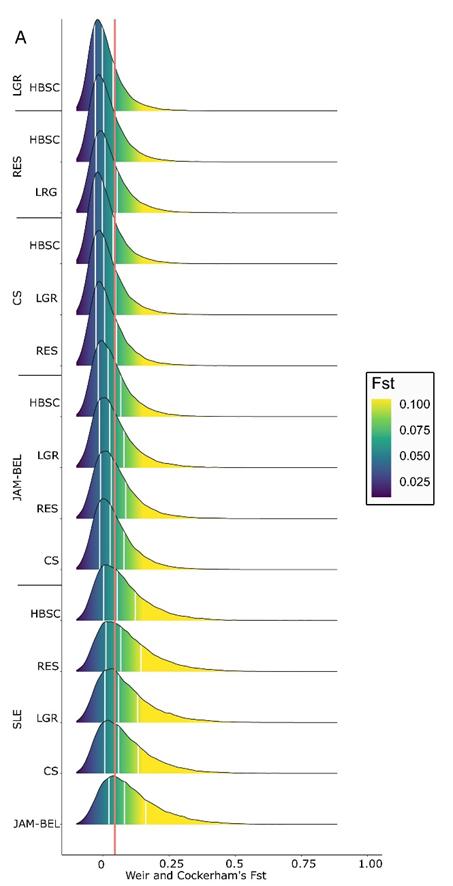


## Figure S9. Density distribution plots for Weir and Cockerham’s F_ST_ measurements for eastern Canada beluga using lcWGS. Estimates were made using 50 kbp windows. White lines indicate quantiles and the orange vertical line indicates overall mean F_ST_.


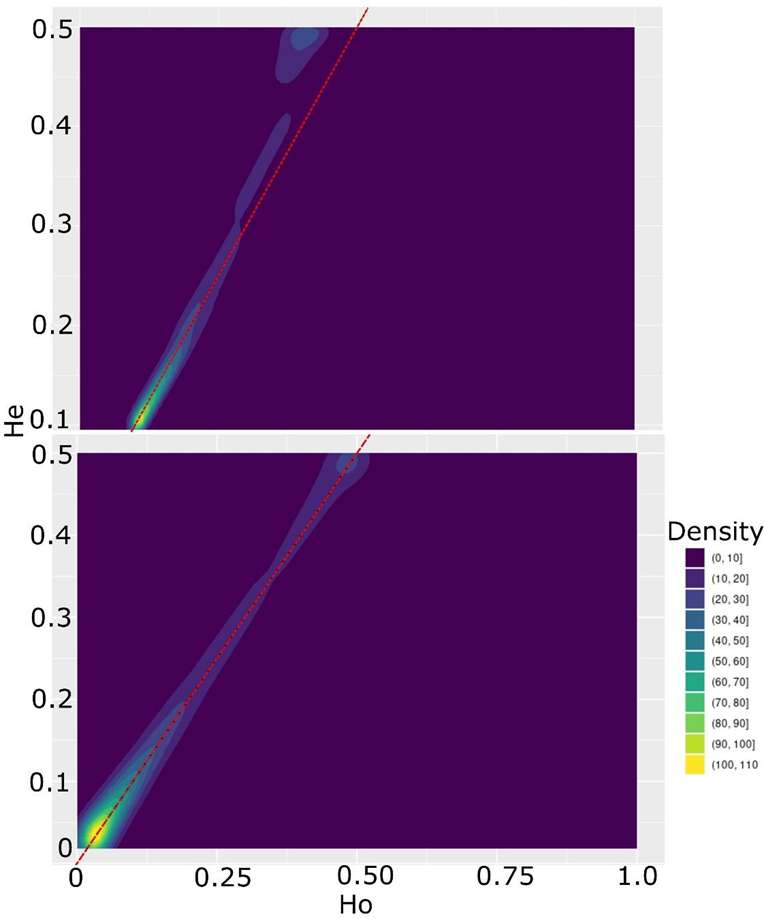


## Figure S10. Density of observed (Ho) versus expected (He) site heterozygosity values in lcWGS (above) and ddRADseq (below) datasets for beluga tissue collected between 1989 and 2019 in eastern Canada. Note He is cut off from the lcWGS panel given the 0.05 MAF filter was applied prior to this analysis.


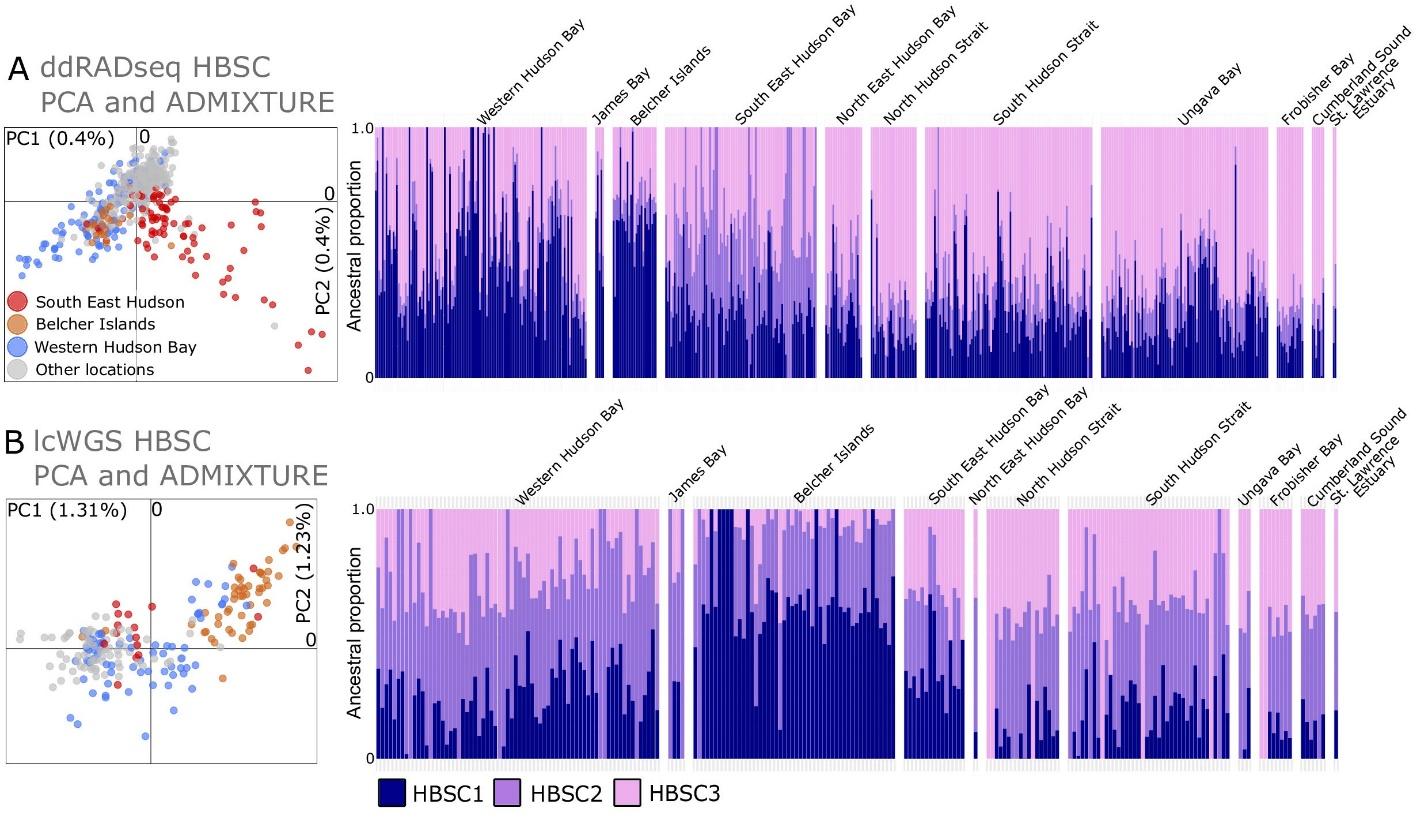


## Figure S11. Substructure results of HBSC belugas. Panels represent ADMIXTURE (K = 3) or Principal Component Analyses (PCAs) for ddRADseq (A) and lcWGS datasets (B).


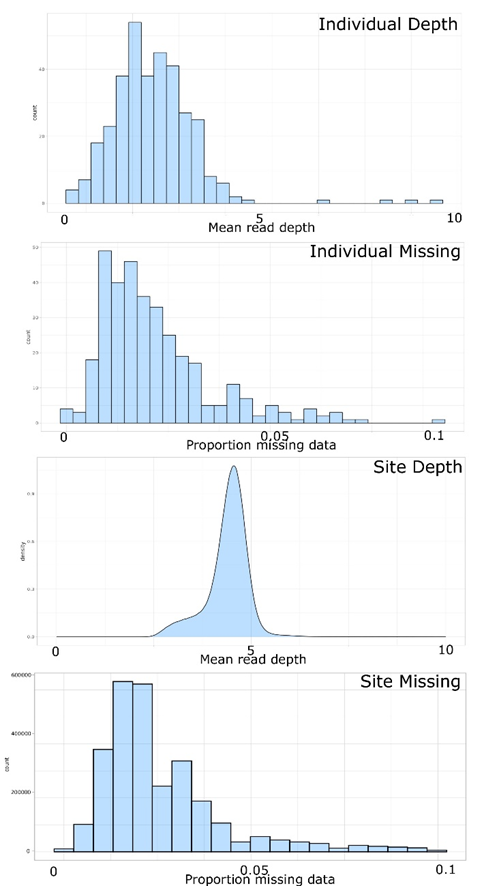


## Figure S12. lcWGS SNPs dataset statistics in eastern Canada beluga harvested, live-biopsied, or found dead in the Hudson Bay and surrounding areas, St. Lawrence estuary and gulf, and Resolute Bay between 1989 and 2019, including missingness and read depth for lcWGS.

# References

Andrews, S. (2010). *FastQC: a quality control tool for high throughput sequence data* (Version 0.12.0) [Computer software]. https://www.bioinformatics.babraham.ac.uk/projects/fastqc/

Bolger, A. M., Lohse, M., & Usadel, B. (2014). Trimmomatic: A flexible trimmer for Illumina sequence data. *Bioinformatics*, *30*(15), 2114–2120. https://doi.org/10.1093/bioinformatics/btu170

Bringloe, T. T., & Parent, G. J. (2023). Contrasting new and available reference genomes to highlight uncertainties in assemblies and areas for future improvement: An example with monodontid species. *BMC Genomics*, *24*(1), 693. https://doi.org/10.1186/s12864-023-09779-3

Capblancq, T., Luu, K., Blum, M. G. B., & Bazin, E. (2018). Evaluation of redundancy analysis to identify signatures of local adaptation. *Molecular Ecology Resources*, *18*(6), 1223–1233. https://doi.org/10.1111/1755-0998.12906

Catchen, J., Hohenlohe, P. A., Bassham, S., Amores, A., & Cresko, W. A. (2013). Stacks: An analysis tool set for population genomics. *Molecular Ecology*, *22*(11), 3124–3140. https://doi.org/10.1111/mec.12354

Danecek, P., Auton, A., Abecasis, G., Albers, C. A., Banks, E., DePristo, M. A., Handsaker, R. E., Lunter, G., Marth, G. T., Sherry, S. T., McVean, G., Durbin, R., & 1000 Genomes Project Analysis Group. (2011). The variant call format and VCFtools. *Bioinformatics*, *27*(15), 2156–2158. https://doi.org/10.1093/bioinformatics/btr330

Danecek, P., Bonfield, J. K., Liddle, J., Marshall, J., Ohan, V., Pollard, M. O., Whitwham, A., Keane, T., McCarthy, S. A., Davies, R. M., & Li, H. (2021). Twelve years of SAMtools and BCFtools. *GigaScience*, *10*(2), giab008. https://doi.org/10.1093/gigascience/giab008

Ewels, P., Magnusson, M., Lundin, S., & Käller, M. (2016). MultiQC: Summarize analysis results for multiple tools and samples in a single report. *Bioinformatics*, *32*(19), 3047–3048. https://doi.org/10.1093/bioinformatics/btw354

Knaus, B. J., & Grünwald, N. J. (2017). vcfr: A package to manipulate and visualize variant call format data in R. *Molecular Ecology Resources*, *17*(1), 44–53. https://doi.org/10.1111/1755-0998.12549

Langmead, B., & Salzberg, S. L. (2012). Fast gapped-read alignment with Bowtie 2. *Nature Methods*, *9*(4), 357–359. https://doi.org/10.1038/nmeth.1923

Li, H. (2013). *Aligning sequence reads, clone sequences and assembly contigs with BWA-MEM* (No. arXiv:1303.3997). arXiv. http://arxiv.org/abs/1303.3997

Li, H., & Durbin, R. (2010). Fast and accurate long-read alignment with Burrows–Wheeler transform. *Bioinformatics*, *26*(5), 589–595. https://doi.org/10.1093/bioinformatics/btp698

Manichaikul, A., Mychaleckyj, J. C., Rich, S. S., Daly, K., Sale, M., & Chen, W.-M. (2010). Robust relationship inference in genome-wide association studies. *Bioinformatics*, *26*(22), 2867–2873. https://doi.org/10.1093/bioinformatics/btq559

Oksanen, J., Simpson, G., Blanchet, F. G., Kindt, R., Legendre, P., Minchin, P., O’Hara, R. B., Solymos, P., Srevens, M., Szoecs, E., Wagner, H., Barbour, M., Bedward, M., Bolker, B., Bocard, D., Carvalho, G., Chirico, M., De Caceres, M., Durand, S., … Weedon, J. (2022). *vegan: Community Ecology Package* (Version R package version 2.6-4) [Computer software]. https://CRAN.R-project.org/package=vegan

O’Leary, S. J., Puritz, J. B., Willis, S. C., Hollenbeck, C. M., & Portnoy, D. S. (2018). These aren’t the loci you’re looking for: Principles of effective SNP filtering for molecular ecologists. *Molecular Ecology*, *27*(16), 3193–3206. https://doi.org/10.1111/mec.14792

Parent, G. J., Montana, L., Bonnet, C., Parent, E., Sauvé, C., St-Pierre, A. P., Watt, C. A., & Hammill, M. O. (2024). Genetic monitoring program for beluga (*Delphinapterus leucas*) harvested in the Nunavik and Nunavut (Belcher Islands) regions. *Canadian Technical Report of Fisheries and Aquatic Sciences*, *3643*.

Purcell, S., Neale, B., Todd-Brown, K., Thomas, L., Ferreira, M. A. R., Bender, D., Maller, J., Sklar, P., De Bakker, P. I. W., Daly, M. J., & Sham, P. C. (2007). PLINK: A Tool Set for Whole-Genome Association and Population-Based Linkage Analyses. *The American Journal of Human Genetics*, *81*(3), 559–575. https://doi.org/10.1086/519795

R Core Team. (2024). *R: A language and environment for statistical computing* (Version 4.3.3) [Computer software]. R Foundation for Statistical Computing. https://www.R-project.org/

Rochette, N. C., & Catchen, J. M. (2017). Deriving genotypes from RAD-seq short-read data using Stacks. *Nature Protocols*, *12*(12), 2640–2659. https://doi.org/10.1038/nprot.2017.123

Rochette, N. C., Rivera‐Colón, A. G., & Catchen, J. M. (2019). Stacks 2: Analytical methods for paired‐end sequencing improve RADseq‐based population genomics. *Molecular Ecology*, *28*(21), 4737–4754. https://doi.org/10.1111/mec.15253
